# Engineering the Electron Relay in [FeFe]-Hydrogenase Enhances Electrocatalytic H_2_ Evolution

**DOI:** 10.1021/acscatal.5c03665

**Published:** 2025-11-05

**Authors:** Tin Pou Lai, William K. Myers, Stephen B. Carr, Miguel A. Ramirez, Kylie A. Vincent, Simone Morra, Patricia Rodríguez-Maciá

**Affiliations:** † Department of Chemistry, Inorganic Chemistry Laboratory, 6396 University of Oxford, South Parks Road, Oxford OX1 3QR, U.K.; ‡ Research Complex at Harwell, Rutherford Appleton Laboratory, Harwell Campus, Didcot OX11 0QX, U.K.; § Faculty of Engineering, 6123 University of Nottingham, Coates Building, University Park, Nottingham NG7 2RD, U.K.; ∥ School of Chemistry and Leicester Institute for Structural and Chemical Biology, 4488 University of Leicester, University Road, Leicester LE1 7RH, U.K.

**Keywords:** H_2_-conversion, metalloenzymes, electrocatalysis, catalytic bias, truncations, inhibitor sensitivity

## Abstract

H_2_ is an ideal energy vector, but catalysts for its clean production from water are inefficient or expensive. [FeFe]-hydrogenases are the most active H_2_-converting catalysts in nature, using a unique organometallic active site finely tuned by the protein matrix. M3 type [FeFe]-hydrogenases from *Clostridium pasteurianum* and *Clostridium acetobutylicum* are exceptionally active for H_2_ production, and less O_2_ sensitive than most other types of [FeFe]-hydrogenases, making them attractive targets for biotechnology. However, they are more challenging to work with because of their large size and the number of iron–sulfur clusters. Here, the [FeFe]-hydrogenase from *C. acetobutylicum* was systematically engineered to truncate each iron–sulfur-containing region of the F-domain, yielding smaller and easier-to-produce catalytic systems. Detailed characterization revealed that these variants retain high electrocatalytic performance and other essential properties of the natural enzyme.

## Graphical abstract

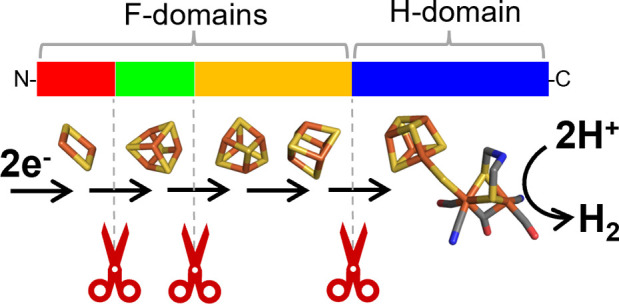

As one of the most efficient H_2_-converting catalysts, [FeFe]-hydrogenases catalyze one of the simplest, but most important chemical reactions, the interconversion of dihydrogen with protons and electrons (H_2_ ⇌ 2H^+^ + 2e^–^). As such, these enzymes provide inspiration for green H_2_ production, which is essential for future sustainable energy systems.^1^ They catalyze this reaction at very high rates (up to ∼10^4^ s^–1^) under ambient conditions in a reversible manner and with minimal overpotential requirement.^2^ All [FeFe]-hydrogenases contain a unique active site, the H-cluster (Figure 1A), composed of a canonical [4Fe–4S] cluster [4Fe–4S]_H_ covalently bound via S-Cys to a diiron subcluster [2Fe]_H_ that contains an open coordination site at the distal Fe (Fe_d_). A critical feature of the H-cluster is the azadithiolate group (ADT), bridging the two Fe ions. The nitrogen atom of ADT can act as either a proton donor or acceptor to facilitate H_2_ evolution and H_2_ oxidation, respectively.^3^ The two Fe ions of [2Fe]_H_ are further coordinated by one terminal CO and CN^–^ ligand each and by a bridging CO. [FeFe]-hydrogenases are highly modular enzymes. The smallest M1 type only contains the H-domain, which harbors the H-cluster. M1 type [FeFe]-hydrogenases are well characterized in green algae, such as *Chlamydomonas reinhardtii* (*Cr*HydA1),^4^ and have been recently detected in archaea.^5^ All other [FeFe]-hydrogenases contain additional accessory domains (F-domains)^6–9^ that host additional iron–sulfur (FeS) clusters, which play an important role in intermolecular electron transfer with redox partners, and intramolecular electron transfer between the H-cluster and the protein surface.^10^ It has also been suggested that the FeS clusters in the F-domain play an important role in making the enzyme more O_2_ resistant, as they can supply the active site with the necessary electrons to reduce O_2_ to water and avoid the formation of reactive oxygen species (ROS), which destroy the H-cluster.^11^ M2 type [FeFe]-hydrogenases contain a single F-domain, with high sequence similarity to bacterial ferredoxins, hosting two [4Fe–4S] clusters (conventionally referred to as FS4A and FS4B, Figure 1B).^12^ Examples of M2 type [FeFe]-hydrogenases are found in *Desulfovibrio desulfuricans* (*Dd*HydAB) and *Megasphaera elsdenii* (*Me*HydA).^13–15^ Larger M3 type [FeFe]-hydrogenases contain two further F-domains (Figure 1B), one harboring a [4Fe–4S] cluster ligated by one histidine and three cysteine residues (FS4C), and the other containing a plant-type ferredoxin [2Fe–2S] cluster (FS2).^16^ Examples of M3 type enzymes are found in *Clostridium pasteurianum* (*Cp*HydA1 or *Cp*I) and *Clostridium acetobutylicum* (*Ca*HydA1).^12,17^

**Figure 1:**
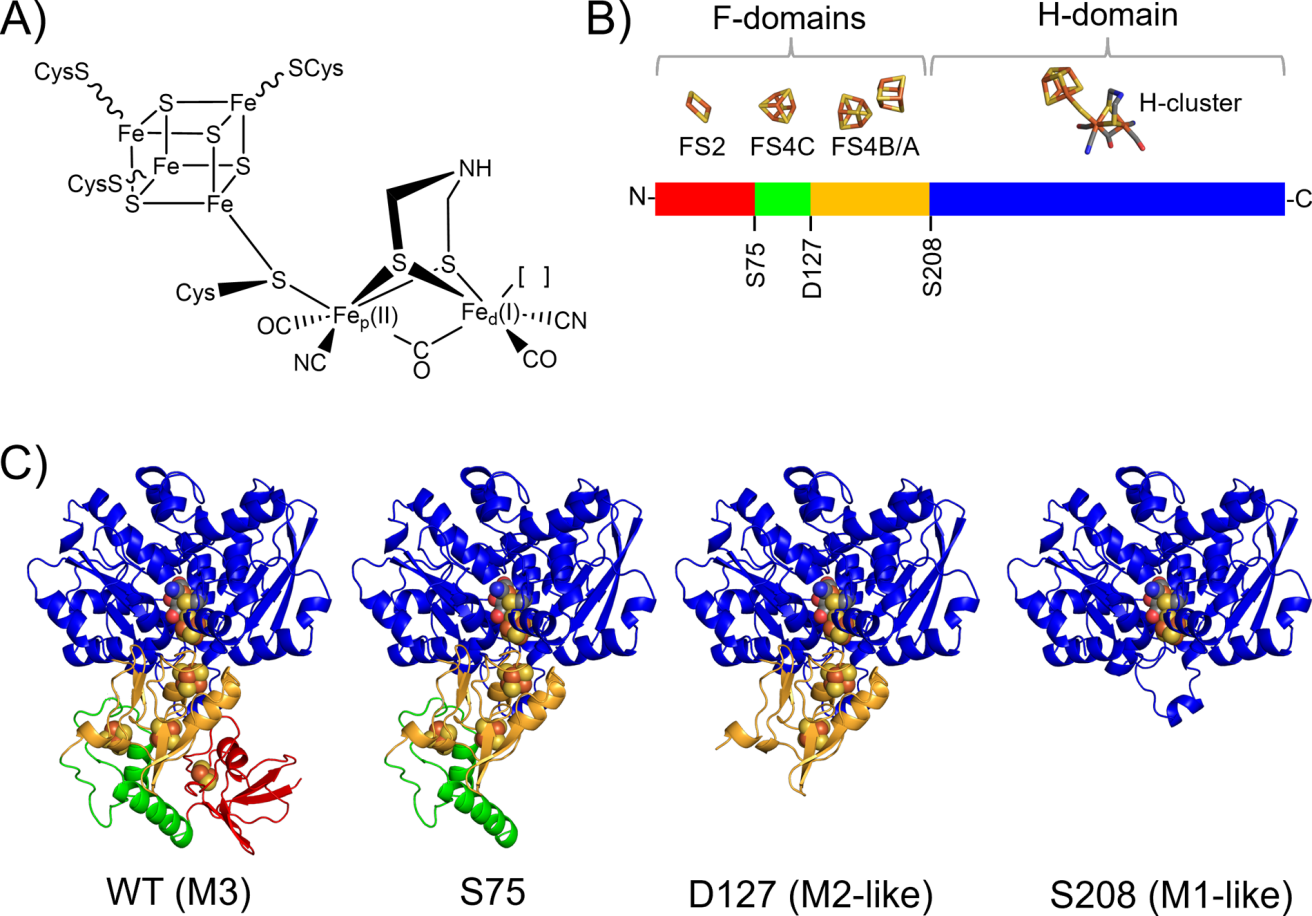
Structural models of the *Ca*HydA1 [FeFe]-hydrogenase truncated variants demonstrate the systematic truncation of the F-domains described herein. The FS2 plant-type [2Fe–2S] domain (red) was removed in a variant that starts at serine 75 (S75); the FS4C histidine-ligated [4Fe–4S] domain (green) was removed in a variant that starts at aspartate 127 (D127) and the FS4A/B bacterial ferredoxin domain (orange) was removed in a variant that starts at serine 208 (S208). The core H-domain containing the active site H-cluster is shown in blue. (A) Schematic representation of the H-cluster in the H_ox_ redox state. (B) Schematic representation of *Ca*HydA1 structure highlighting the residues selected for each truncation. (C) AlphaFold models of the *Ca*HydA1 structure and its truncated variants. The wild-type (WT) *Ca*HydA1 enzyme is the M3-type, while removal of F-domains in the D127 variant makes it the M2-like type (i.e., similar to *Dd*HydAB and *Me*HydA), and the S208 variant is the M1-like type (i.e., similar to *Cr*HydA1).

The role of F-domains in *Ca*HydA1 has been investigated previously by site-directed mutagenesis coupled to spectroscopic, electrochemical, and biochemical assays.^18–23^ Removal of the entire F-domain by N-terminal truncation in *Ca*HydA1 resulted in severe activity loss, but did not alter the O_2_ inactivation kinetics of the enzyme.^20,21^ Likewise, truncation of the whole F-domain in *Me*HydA displayed unaltered O_2_ sensitivity relative to the full-length enzyme, challenging the hypothesis that additional FeS clusters play a main role in O_2_ sensitivity/resistance.^24^ Selectively inactivating the FS4C or the FS2 cluster by site-directed mutagenesis (but maintaining the protein scaffold) of *Ca*HydA1 also resulted in variants with significantly lower catalytic rates, and diminished interactions with the physiological redox partner, highlighting the importance of the FS2 domain.^19^ Studies on the isolated domain hosting the FS4C cluster demonstrated that histidine coordination is crucial in tuning the cluster’s redox potential and electronic properties, influencing its role in electron transfer.^22^ EPR-monitored redox titrations in *Cp*HydA1 have shown a large degree of magnetic coupling between the clusters,^23^ but have proposed that the FS4C domain may be the point of contact with the physiological redox partner, in apparent contrast with other evidence. Recent efforts at exploring the diversity of [FeFe]-hydrogenases have discovered some previously undetected examples of natural O_2_ protection.^25^ For example, an M2-type [FeFe]-hydrogenase from *Clostridium beijerinckii* (*Cb*A5H)^25^ displays a unique O_2_-protection mechanism that has been proposed to be based on a conformational change in the H-domain,^26,27^ while two F-domains appear to control the dimerization state (Zn^2+^-binding SLBB domain) and electron transfer (FS4A/FS4B domain),^28^ and their removal by truncation results in loss of activity.^29^ Fusion of *Cb*HydA1 (an enzyme identical to *Cb*A5H except for 2 nonconserved residues) with the photosystem I subunit PsaE influenced the catalytic activity of the enzyme, suggesting that altering the modular structure of [FeFe]-hydrogenases does modulate their function.^30^

In order to obtain a more granular and detailed characterization of the specific role of each individual cluster within the F-domains of *Ca*HydA1, here we report on the systematic and stepwise truncation of the enzyme. Each F-domain was removed individually, generating a set of three truncated proteins (Figure 1B,C). These are functionally and spectroscopically characterized and compared with those of the wild-type (WT) enzyme, producing a comprehensive investigation into the effect of each F-domain on the catalytic properties of *Ca*HydA1.

## Results

### Influence of the FeS Cluster Domains on the Catalytic Bias and Overpotential

WT and truncated variants were produced recombinantly in *Escherichia coli* at high yield as apo-enzymes containing all iron–sulfur clusters but lacking the [2Fe] subcluster of the H-cluster (Figure S1 and Table S1). It is notable that the shortest variants of *Ca*HydA1 were readily expressed and purified at yields higher than those of the WT, up to 4-fold for D127 and 5.6-fold for S208 (Table S1). All forms contained the expected iron–sulfur cluster content (Figures S3, S4 and Table S2) and showed no major differences in stability with respect to WT. Active enzymes (i.e., holo-enzymes) were produced via artificial maturation.^32,33^ Next, the catalytic activity of the WT and truncated variants was investigated by protein film electrochemistry (PFE) at various pH values (pH range 6–8, Figure S5). The cyclic voltammogram at pH 7 is shown in Figure 2A. *Ca*HydA1 WT (Figure 2A, black trace) shows reversible behavior (cutting the zero-current line at a steep angle at the thermodynamic equilibrium potential), displaying high electrocatalytic currents in both directions. This behavior is consistent with previous electrochemical studies of *Ca*HydA1.^34,35^ Although high potential inactivation of WT *Ca*HydA1 has been observed before under different conditions,^18,23^ there is no evidence for inactivation at high potentials at standard scan rates under the conditions used here (Figure 2A at 20 mV/s, and Figure S7 at 5 mV/s). Interestingly, all truncated variants exhibit similar catalytic behavior to WT (Figures 2A and S6), with each variant showing bidirectional catalysis with a strong bias toward H_2_ evolution. As proposed before,^10^ we hypothesize that the F-domain is the site of interaction between the enzyme and the electrode; therefore, the truncations are likely to affect the interfacial electron transfer rate. Since each truncated variant is smaller in size and likely displays different surface-charge distributions (Figure S13), we also expect that the electroactive coverage of the WT and truncated variants will differ. Regardless of the electroactive coverage, it is clear that when adsorbed on an electrode surface, the truncated proteins exhibit very high catalytic activities reaching current densities of several mA/cm^2^, in some cases exceeding the WT, and, very importantly, without introducing any overpotential for catalysis in either direction. Although it is difficult to be completely consistent during electrode preparation due to inherent variabilities, we observed very similar currents and CV shapes for duplicate protein films (Figure S9). Interestingly, all three truncated variants show some degree of high potential inactivation-reductive reactivation, particularly at slow scan rates, while this is not obvious for the WT enzyme under the same conditions (Figure S7 and Table S4 for calculated *E*_switch_). Figure S8 and Table S3 showed the observed *j*_red_/*j*_ox_ ratios (where *j* is the current density, i.e., electrocatalytic current/electrode area) at pH 7, calculated following reported procedures,^36^ allowing estimation of the catalytic bias for each variant. While *j*_red_/*j*_ox_ for WT at ± 100 mV is ≈1.3, at the same overpotential, *j*_red_/*j*_ox_ is ≈3 for all the truncated variants, indicating a stronger preference for H_2_ evolution. This would be consistent with the most exposed cluster in *Ca*HydA1 (the [2Fe–2S] cluster) having a more positive potential than that of the other clusters.

**Figure 2:**
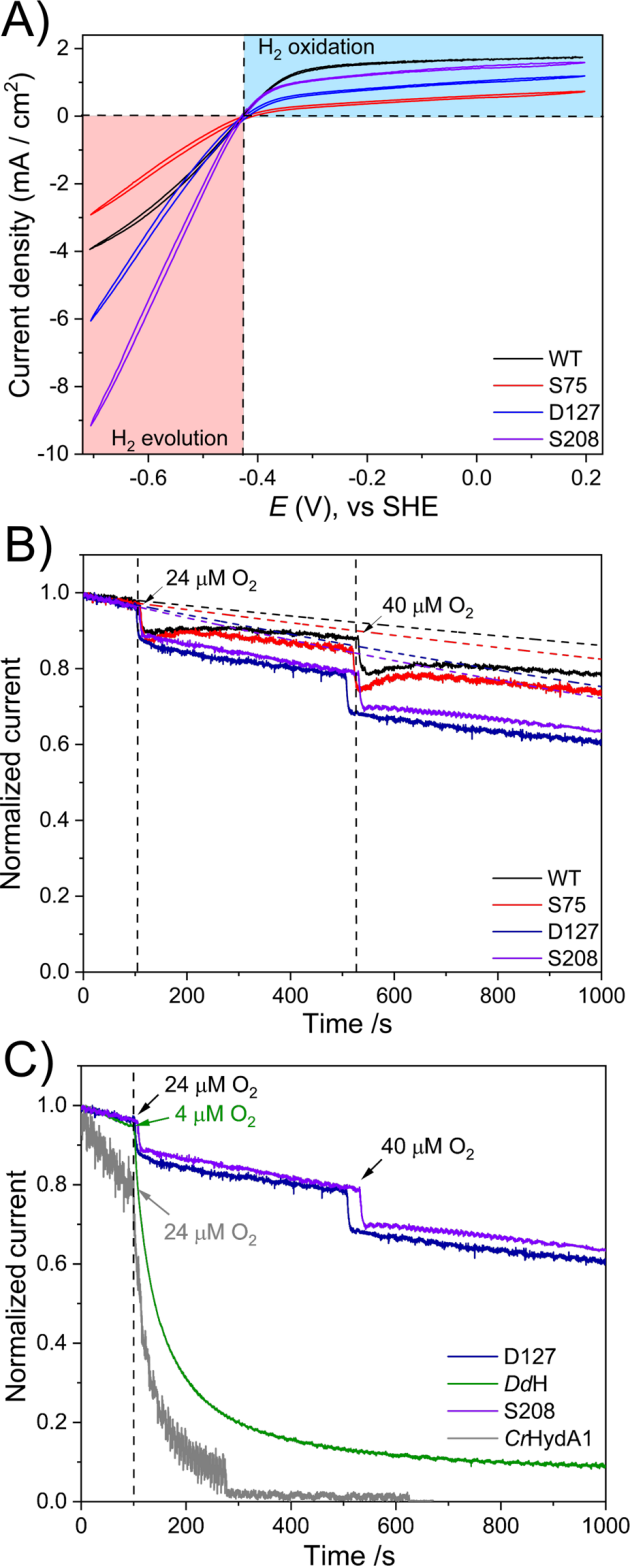
(A) Cyclic voltammograms of WT and truncated *Ca*HydA1 [FeFe]-hydrogenases adsorbed onto a pyrolytic graphite electrode at pH 7, room temperature, constant flow of 100% H_2_ (1 L/min), 2000 rpm, and 20 mV/s scan rate. The horizontal dashed line represents the zero current, and the vertical dashed line indicates the thermodynamic potential for the 2H^+^/H_2_ couple at the given pH. (B) Normalized chronoamperogram showing the aerobic inactivation of WT *Ca*HydA1 and its truncated variants. The enzymes were adsorbed onto a rotating pyrolytic graphite electrode and poised at +40 mV versus SHE under a constant flow of 100% H_2_ to flush out the O_2_. The H_2_ oxidation current was monitored vs time following the injection of small amounts of O_2_ in the electrochemical cell. The solid lines are the experimental data, and the dotted lines are the projected current baselines accounting for film loss,^31^ extrapolated from the anaerobic part of the data (recorded in the first 100 s) by fitting to an exponential function. Vertical dashed lines indicate the points where O_2_ was added to the electrochemical cell. (C) Aerobic inactivation comparison between M2-type like D127, and the enzyme *Dd*HydAB and M1-type like S208 and *Cr*HydA1. Enzymes were absorbed onto a PGE, and the potential was poised at +40 mV vs SHE under a 100% H_2_ atmosphere. The H_2_ oxidation current was monitored versus time following the injection of small amounts of air-saturated buffer into the electrochemical cell. For (B) and (C), the data were normalized in the range of [1,0] using OriginPro software by setting the initial current at *t* = 0 to 1 and the baseline (zero current) to 0.

### O_2_ and CO Inhibition of H_2_ Oxidation

The inactivation of the WT and truncated variants by O_2_ and CO (Figures 2B, S10 and S12) was studied by chronoamperometry.^34^ In order to monitor any effect of O_2_ on the electrocatalytic H_2_ oxidation by each enzyme, the electrode potential was set to +40 mV (a potential where the enzymes are oxidizing H_2_ but more positive than the reduction potential of the O_2_ on graphite to avoid direct reduction of the O_2_ at the electrode surface). The injection of O_2_ caused an immediate drop in electrocatalytic current for all of the proteins. In both WT and truncated variants, ≈10% of activity was lost. WT enzyme shows some recovery of activity (as previously reported)^18^ as O_2_ is displaced from the electrochemical cell by the flow of H_2_. The S75 variant displays a similar degree of recovery to WT, whereas loss of activity in the D127 and S208 variants appears to be irreversible after O_2_ exposure. Interestingly, the behavior of the D127 and S208 variants is in stark contrast to their respective counterparts, M2-type *Dd*HydAB and M1-type *Cr*HydA1 [FeFe]-hydrogenases (Figures 2C and S11). *Ca*HydA1 truncated variants are much more resistant to O_2_ than their native counterparts containing the same number of additional FeS clusters. This suggests that the O_2_ resistance of *Ca*HydA1 is an intrinsic property of the H-domain (e.g., gas channels, or active site characteristics) rather than controlled by the F-domains (FS2, FS4C). CO inactivation was also studied by chronoamperometry (Figure S12), setting the potential at −160 mV, a potential where the enzymes are oxidizing H_2_, to monitor the response of the H_2_ oxidation electrocatalytic current to the competitive inhibitor CO. The extent of inhibition was similar for all the enzymes, and appears to be fully reversible, with current recovering after CO is flushed away with H_2_.

### Solution Activity Assays with Artificial and Physiological Redox Partners

In apparent contrast with PFE data, turnover rates measured by solution activity assays for the truncated *Ca*HydA1 [FeFe]-hydrogenases are drastically lower than those of the WT for both H_2_ oxidation and evolution (Figure 3). Assays using methyl viologen (MV) as an artificial electron partner revealed that the activity of the truncated enzyme increases with the degree of truncation, i.e., the smaller S208 variant exhibits the highest specific activity in both reaction directions (Figure 3A and Table S5). Discrepancy between PFE activity data and assays performed with soluble artificial redox partners may be due to changes in the charge distribution on the surface of the enzyme, which would affect interaction with the redox partner more severely (Figure S13).

**Figure 3:**
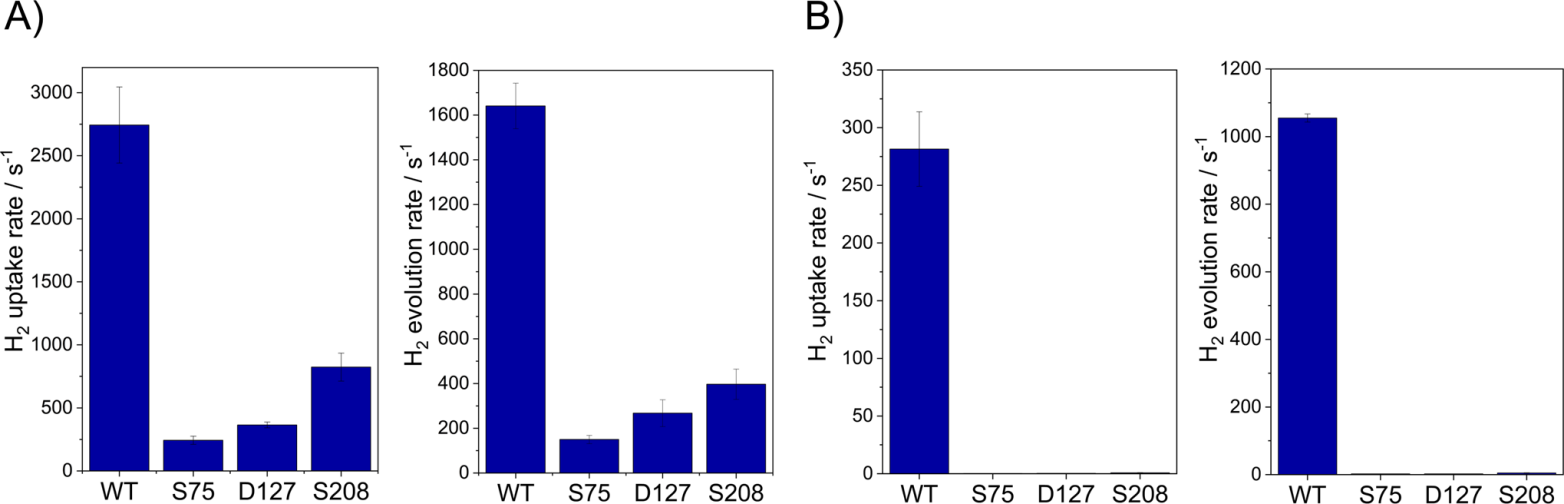
(A) Solution activity assays of WT *Ca*HydA1 and truncated variants. The H_2_ uptake (oxidation) rate was measured by following the reduction of methyl viologen by the enzyme in a H_2_-saturated solution. The H_2_ evolution rate was measured by gas chromatography (GC) using methyl viologen as an electron donor. All rates are measured in 100 mM Tris-HCl, 150 mM NaCl, pH 8, with 10 mM methyl viologen, 37 °C. Rates are given in s^–1^, as U/mg would be inaccurate due to a change in enzyme size. (B) *S*olution activity assays of WT *Ca*HydA1 and truncated variants using the native redox partner, ferredoxin (*Ca*Fd) following the same methodology.^17^ Rates used to plot this figure are presented in Supplementary Tables S5 and S6.

There is a debate about which FeS cluster provides the physiological entry point of electrons in M3-type [FeFe]-hydrogenases. This is important because the properties of the distal cluster (redox potential, spin state, spatial orientation) are crucial to controlling intermolecular and intramolecular electron movement to enable fast electron transfer between electron donor/acceptor molecules and the FeS cluster relay for coupling to reversible H_2_ activation at the active site.^22^ Solution activity assays with the native redox partner of *Ca*HydA1, ferredoxin CAC0303 (*Ca*Fd)^17,37–39^ show significant activity for either H_2_ oxidation or production only for WT *Ca*HydA1 (Figure 3B and Table S6). The loss of detectable activity for all truncated variants when *Ca*Fd is used as a redox partner points to the [2Fe–2S] cluster (FS2) containing domain as the ferredoxin binding site. This is supported by a protein–protein docking study, which revealed the [2Fe-2S] domain of *Ca*HydA1 as the most likely docking site for *Ca*Fd (Figure S14), in accordance with a previous site-directed mutagenesis study.^19^ On the other hand, previous spectroscopic work on *Cp*HydA1 suggested that the ferredoxin binding site is on the His-ligated [4Fe–4S] (FS4C) domain,^23^ and we hypothesize that such a difference is due to significant sequence variations within the F-domains that may determine a completely different binding preference (Figure S15)

### Infrared Spectroscopy

Infrared (IR) spectroscopy was used to characterize the H-cluster in the WT and truncated *Ca*HydA1 variants, allowing the characterization of different redox states, such as the oxidized H_ox_, the 1-electron reduced H_red_/H_red_H^+^, the 2-electron reduced H_hyd_/H_sred_H^+^, and the CO-inhibited H_ox_-CO.^40,41^ All WT spectra (Figures 4 and S16) are well aligned with previous literature,^42,43^ suggesting that the artificial maturation method used here results in preparations that are equivalent to those obtained by native maturases but with increased yields and purity, as previously reported for other [FeFe]-hydrogenases.^32,33^ The “as isolated” WT sample maturated with ADT displays five major peaks that can be assigned to the H_ox_ state (Figure 4A): 1801 (bridging CO), 1969, 1946 (terminal CO), and 2082, 2070 cm^–1^ (CN^–^). All truncated proteins have nearly identical IR spectra (Figure 4A), but they differ from the WT as most peaks are blue-shifted (i.e., to higher wavenumbers) by 5 cm^–1^, with the exception of the peak for the bridging CO, which is red-shifted by 4 cm^–1^. The sensitivity of the CO and CN^–^ band positions to changes in electron density at a metal center means that these spectra provide insight into the electronic environment at the hydrogenase active site. Overall, the spectra demonstrate that the electronic environment of the H-cluster is consistent between truncated variants but differs slightly from the WT enzyme, suggesting a long-range effect of the FeS clusters in the F-domain on the H-cluster and/or conformational gating of protein–protein binding at their interface.

**Figure 4:**
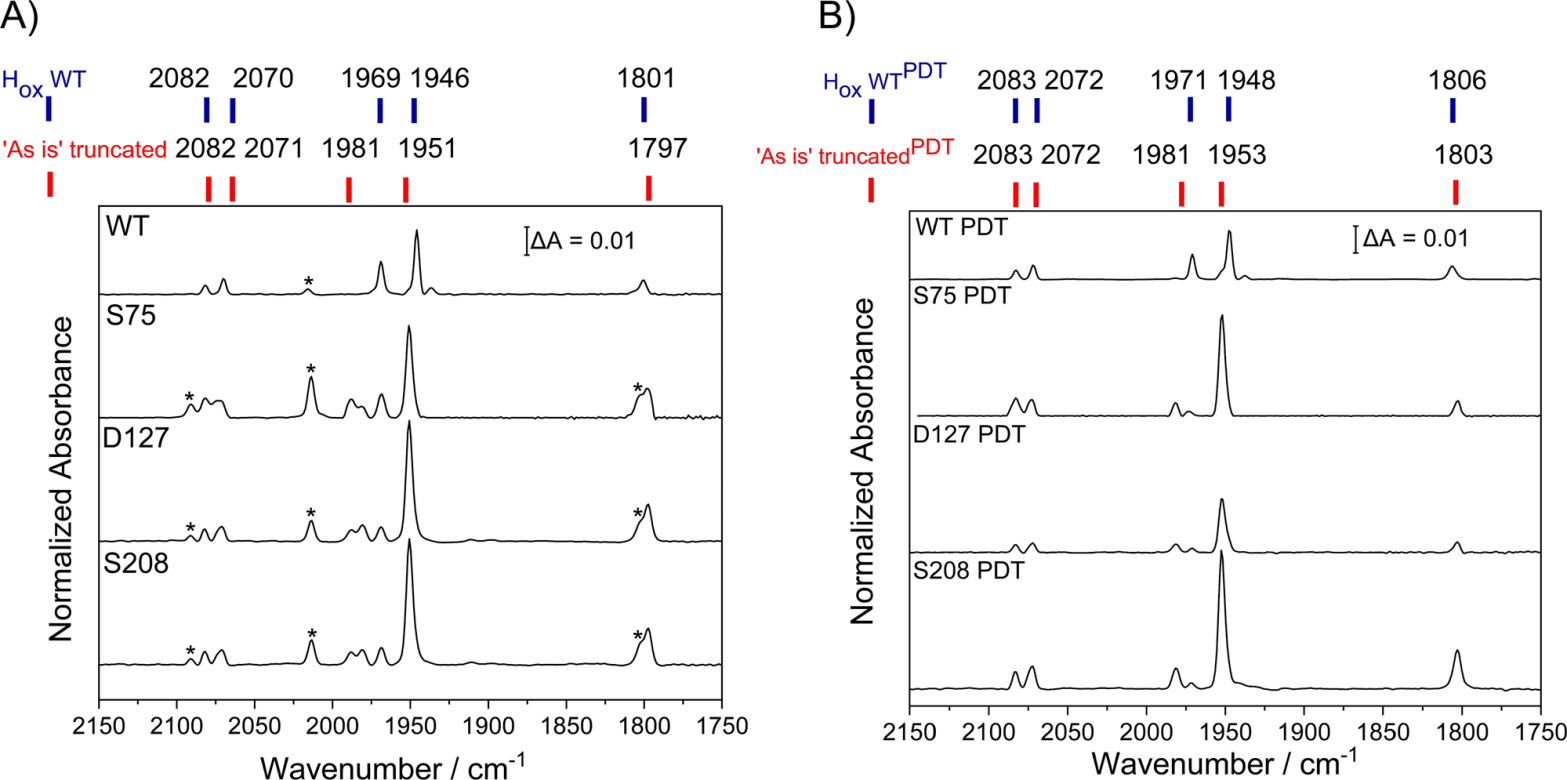
FTIR spectra of “as isolated” WT and truncated *Ca*HydA1 proteins at pH 8. (A) Maturated with the native-like cofactor ADT. (B) Maturated with the synthetic active-site analogue PDT to give a non-native H-cluster. The main observed redox states are highlighted in blue and red at the top. Sample concentration is ≈1–2 mM, prepared in a N_2_ glovebox, spectra are an average of 512 scans measured at room temperature and at 2 cm^–1^ resolution. Additional minor bands, marked by an asterisk, correspond to the H_ox_-CO state, likely formed by the so-called “cannibalization” process.^44^

Taking advantage of artificial maturation, we maturated *Ca*HydA1 and its variants with the synthetic non-native H-cluster analogue propane 1,3-dithiolate (PDT) (Figure 4B), allowing for detailed insights into not only the [2Fe]_H_ subcluster but also the [4Fe–4S]_H_ cluster. [FeFe]-hydrogenases maturated with PDT yield inactive protein since the CH_2_ bridgehead-group of the PDT cofactor cannot participate in proton transfer, shifting focus to the [4Fe–4S]_H_.^45^ The spectrum of “as isolated” WT-PDT displays five peaks with the main band at 1948 cm^–1^ (2 cm^–1^ higher than WT-ADT). With PDT, the three truncated variants again display identical spectra to each other, but are blue-shifted relative to WT. The well-characterized CO-inhibited state (H_ox_-CO) in WT-ADT displayed a similar pattern of peak shifts: blue-shifted for all terminal CN^–^ and CO bands, and red-shifted for the bridging CO band (Figure S16A). Spectra of reduced samples were investigated by the addition of 10 mM sodium dithionite under a H_2_ atmosphere (100% H_2_), yielding a mixture of states in all the proteins, including H_red_H^+^, H_hyd_, and H_ox_ (from reoxidation) (Figure S16). In contrast to the H_ox_ state, the main bands for H_red_H^+^ are identical for WT and truncated variants (Figure S16 and Table S7). Interestingly, peaks that are reminiscent of the H_hyd_ state are visible in the spectra of all the truncated variants but not in the WT spectrum. Conversely, small bands reminiscent of the H_red_ and H_sred_H^+^ states are observed only in the spectrum for the WT. This suggests that the redox potential of the truncated variants differs from that of the WT, making protonation of the active site easier in the mutants and thus resulting in altered redox equilibria between states.

### EPR Spectroscopy

The H_ox_ and H_ox_-CO states in [FeFe]-hydrogenases are paramagnetic (*S* = 1/2).^46^ Similarly to IR spectra, EPR spectra of the artificially maturated WT (Figure 5) closely resemble those previously reported for preparations obtained with native maturases.^42^ The EPR spectrum for WT (Figure 5A) is dominated by a rhombic signal with g values *g*_1_ = 2.096, *g*_2_ = 2.0395, *g*_3_ = 2.0005, which agree with those reported for the H_ox_ state.^42^ The signature at higher magnetic field, ca. 347 mT, has been attributed to the reduced [2Fe–2S] center FS2 in WT *Ca*HydA1, and its absence in the truncated variants further confirms this assignment (see below).^42,47–49^ There is also a contribution from an axial signal, which we assign to H_ox_-CO (Figure 5B). The WT H_ox_ state has a second component at 10% with a lower isotropic *g*-value, *g*_iso_ = 2.042, than the principal component with *g*_iso_ = 2.045 (Table S8). Two forms of H_ox_ differing slightly in their *g*-values, termed H_ox_(1) and H_ox_(2), were previously detected in the *Cb*HydA1 [FeFe]-hydrogenase and determined to be due to subtle structural changes around the [4Fe–4S]_H_ cluster.^50^ Truncated variants generally follow the lower *g*_iso_ value, but in D127, the H_ox_ state contains both components reversed in concentration compared to that of the WT (Figure S17). The S75 variant has a *g*_iso_ of 2.041. These small shifts in *g*_iso_ imply changes in FeS cluster coupling, but the local geometry of the H-cluster is largely unaltered in the truncated variants. Presence of a minor component in the WT is also seen in the EPR spectrum of the pure H_ox_-CO state (Figure 5B). This spectrum has characteristic g-values of *g*_1_ = 2.075, *g*_2_ = 2.007, and *g*_3_ = 2.007. It agrees well with the previous H_ox_-CO EPR spectrum reported for WT *Ca*HydA1 and other [FeFe]-hydrogenase systems.^42^ However, satisfactory simulation of the WT H_ox_-CO state also requires incorporation of a second component at ca. 10% relative intensity with *g*_∥_ = 2.0225, which cannot be explained with the small [2Fe–2S]^+^ signal fraction (Figure S18). As with the WT H_ox_ state, the truncated variants exhibit signals matching the minor component of the WT with a *g*_∥_ between 2.0225 and 2.020 (Table S8). However, in this case, none of the variants exhibit the H_ox_-CO component, which is significant in the WT sample. The EPR spectrum reported for the H_ox_-CO state in the truncated version of *Me*HydA is very similar to the WT minor component of our truncated variants, with almost identical g values (*g*_1_ = 2.020, *g*_2_ = 2.009, *g*_3_ = 2.008) despite the fact that the IR spectra of the H_ox_-CO states in *Me*HydA and *Ca*HydA1 truncated variants are significantly different.^24^ Heghmanns and co-workers reported an unknown radical signal (R.ox) in the oxidized state of the oxygen-protected [FeFe]-hydrogenase from *Clostridium beijerinckii* (*Cb*A5H),^51^ which was subsequently suggested to represent an alternative form of H_ox_-CO, named H_ox_-CO­(1) in *Cb*HydA1.^50^ Interestingly, the EPR spectrum of our truncated variants (*g* values: *g*_1_ = 2.019, *g*_2_ = 2.010, *g*_3_ = 2.006) is almost identical to the previously reported R.ox and H_ox_-CO­(1). The EPR spectrum of R.ox exhibits a nearly isotropic signal at *g* = 2.010. This state could be obtained in *Cb*A5H after the enzyme had reacted with O_2_ or oxidants.

**Figure 5:**
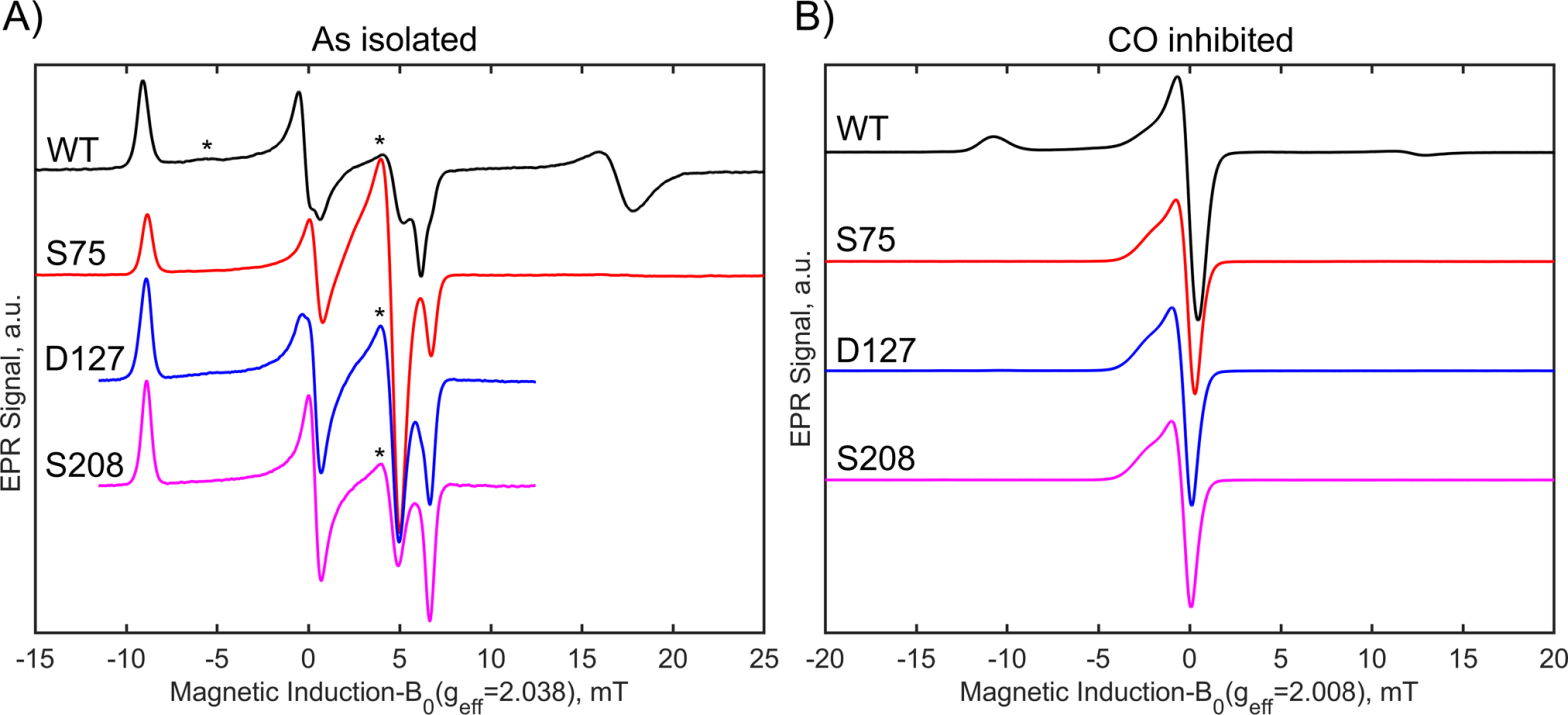
X-band CW-EPR spectra of 200 μM [FeFe]-hydrogenase in the “as isolated” state (A) and H_ox_-CO state (B). The traces are wild type, S075, D127, and S208, and truncated proteins, respectively, at pH 8, as acquisition values in the Methods section. Features marked with an asterisk correspond to the H_ox_-CO state. Measurement conditions are in Methods.

## Discussion

This work demonstrates that the M3 type [FeFe]-hydrogenase *Ca*HydA1 can be engineered by a systematic and stepwise truncation of the F-domains to investigate the specific role of the FeS clusters in the enzyme’s catalytic properties, and produced variants that performed very well when immobilized on an electrode. Production of *Ca*HydA1 and its truncated variants by artificial maturation also allowed for the introduction of modified active sites into the protein. All truncated variants are active for H_2_ conversion and strongly biased toward H_2_ production relative to WT. When adsorbed onto an electrode surface, the variants display higher electrocatalytic currents than the WT enzyme without introducing any overpotential for catalysis. We suggest that the truncations affect the catalytic bias of the enzyme because the catalytic bias may be influenced by the potential of the surface-exposed cluster.^10^ Nevertheless, we cannot exclude influences on the active site, as well. Previous modeling of voltammograms with more complex models showed that multiple catalytic steps may influence the catalytic bias.^52^ However, truncations have a minimal effect on the overpotential and inhibitor sensitivity, suggesting that these properties are predominantly governed by the active site.

Of importance for biotechnological applications, the shorter variants can be produced in much higher yields, with the S208 truncated variant being produced with an almost 6-fold increase in yield compared to WT, which has not been reported before in *Ca*HydA1 truncation studies.^19–21^

Although quantification of electroactive coverage has been performed for *Ca*HydA1 on Au-electrodes by electrochemical scanning tunneling microscopy, these high-resolution microscopy techniques rely on gold electrodes as a flat and conductive surface.^53^ Thus, no suitable method is currently available to determine the exact number of enzyme molecules immobilized on carbon-based electrodes during a PFE experiment, and therefore, an accurate direct comparison between current density (Figure 2A) and reaction rates (Figure 3A) is not possible. We hypothesize that the relative differences are influenced by the different abilities of the variants to bind to the electrode surface *vs* the redox mediators (MV, ferredoxin) and achieve productive electron transfer, due to varying size and surface charge distribution (Figure S13). While accurately dissecting these factors is almost impossible in PFE experiments, we highlight that the smaller enzyme variants D127 and S208 might be beneficial in electrocatalytic applications because their high electrocatalytic currents would directly result in higher H_2_ production per unit area (i.e., current density).

There is an active debate about how the FeS clusters may affect the properties of the active site in hydrogenases.^18,54^ Hexter et al., suggested that the potential of the entry point of electrons in the enzyme (i.e., the most exposed FeS cluster) determines the catalytic bias,^10^ implying that the potential of the most exposed cluster in *Ca*HydA1 should be slightly more negative than the thermodynamic potential for the 2H^+^/H_2_ couple (*E*_*2H^+^/H*_2__). It was proposed by EPR redox titrations that the histidine-ligated cluster FS4C in *Cp*HydA1 has a low reduction potential (*E*_m_< −450 mV vs SHE), but it has not been directly quantified.^23^ In a recent study by Lubner et al., the single histidine ligating the FS4C cluster was mutated to a cysteine.^22^ The potential of the FeS clusters was determined by square wave voltammetry (SWV), and only a small negative shift (∼−65 mV) was observed for the potential of the cluster in this variant.^22^ Our electrochemical data are consistent with the [2Fe–2S] cluster having the most positive potential, supporting the reported redox potential values calculated by redox titrations on *Cp*HydA1, and agreeing with the notion that the His-ligated [4Fe–4S] cluster in these enzymes has a very negative potential.^23^ Interestingly, His-ligated FeS clusters found in other proteins generally have a more positive potential (around −200 mV, vs SHE).^55,56^ Overall, while our data appear to support the role of the surface-exposed cluster as an important factor determining the bias, previous modeling of hydrogenase CVs painted a more nuanced picture.^52^

O_2_ inhibition experiments showed that for the WT and truncated *Ca*HydA1 proteins, even at higher O_2_ concentrations, the catalytic current was only partially inhibited; however, the current recovery after O_2_ exposure is clearly different for WT and truncated variants, and between the truncated variants. This suggests that removal of the F-clusters has a negligible effect on the inactivation by O_2_ but does have a direct influence on the recovery of the current (i.e., reversibility). This indicates that the F-clusters are not directly involved in protecting the enzyme against O_2_ attack (i.e., in the initial O_2_ binding step), but they are involved in providing a “defense” mechanism for the active site to avoid the formation of ROS by providing enough electrons to reduce O_2_ to water.^24,11,57–59^ It is likely that the His-ligated [4Fe–4S] cluster controls the reversibility of oxygen inactivation in *Ca*HydA1, as variants D127 and S208 do not show any recovery. We hypothesize that the enhanced O_2_ resistance of the M3-type [FeFe]-hydrogenases may relate to differences in the gas channel rather than the FeS clusters and that the overpotential is governed by the active site rather than by the FeS clusters in the F-domain.

Likewise, CO inhibition experiments showed reversible inhibition at similar levels across the WT and all of the variants. Since both CO and O_2_ are competitive inhibitors and target the H-cluster, our results imply that the truncations do not significantly alter the gas channels in the enzyme.

The exact entry point of electrons in M3-type [FeFe]-hydrogenases, whether it is the His-ligated [4Fe–4S] or the [2Fe–2S] cluster, is still a topic of debate. Inactivating the individual clusters but maintaining the protein backbone in *Ca*HydA1 resulted in partial activity loss coupled to diminished affinity for ferredoxin, which was more severe when the [2Fe–2S] cluster was targeted suggesting a major role.^19^ Interestingly, altering the His-ligated [4Fe–4S] cluster’s redox potential by mutating the histidine into a cysteine residue, only diminished the specific activity but did not alter affinity, which was proposed to support the entry point at this other domain instead.^22^ Our stepwise truncations of the F-domain allowed us to investigate the binding site of the native redox partner of *Ca*HydA1, ferredoxin *Ca*Fd CAC0303, by probing every cluster in the F-domain, something that has not been possible before. Activity assays with the physiological redox partner and modeling studies imply the binding site for *Ca*Fd is near the [2Fe–2S] cluster domain, since truncated variants lacking this domain display no activity for H_2_ oxidation or production when tested with ferredoxin.

In addition to functional evidence, we show by FTIR and EPR spectroscopy that removing any of the individual F-domains from *Ca*HydA1 has a long-range impact of the electronic properties of the H-cluster. IR spectroscopy demonstrates slight differences in the electronic environment of the H-cluster for WT and truncated proteins. The IR bands in the truncated variants are shifted toward higher wavenumber, indicating a slightly more positive environment at the active site compared to WT.

The EPR spectra and g values of the WT and truncated variants in the H_ox_ state are similar, suggesting that the local geometry of the H-cluster is maintained in all of the systems. However, the H_ox_-CO EPR spectra are markedly different between those of the WT and truncated variants. The H_ox_-CO EPR spectra of our truncated variants look identical to that of H_ox_-CO­(1) EPR spectrum of *Cb*HydA1, which was attributed to a structural change near the [4Fe–4S]_H_ cluster.^50^ We suggest that this may also be the case in the truncated variants, and their unusual H_ox_-CO EPR spectra might be explained by the geometry distortion effect in which the coordination environment of the [4Fe–4S]_H_ cluster is perturbed in the truncated proteins, which in turn alters the energy of the orbitals on [2Fe]_H_. This effect is more pronounced for H_ox_-CO relative to H_ox_ because the coupling (*J*) between the [4Fe–4S]_H_ cluster and [2Fe]_H_ subclusters is stronger in H_ox_-CO.^50^

In conclusion, we have demonstrated that truncation of the F-domains in *Ca*HydA1 enhances purification yields significantly, almost 6-fold for the smallest variant. Overall, our results highlight the potential of these truncated variants for biotechnological applications and shine light on the role of the FeS clusters on the catalytic activity, O_2_ sensitivity, and catalytic bias of [FeFe]-hydrogenases.

## Methods

### Preparation of WT Apo-*Ca*HydA1

The *Ca*HydA1 gene with a C-terminal StrepTagII sequence was PCR amplified from plasmid pCaAE^20^ and cloned into a pET-21a vector between NdeI/XhoI sites using the NEBuilder HiFi assembly cloning kit (NEB). The resulting plasmid was verified by DNA sequencing and transformed into *E. coli* BL21­(DE3)­Δ*iscR*.^60^ Bacteria were grown in Lysogeny Broth (LB) media (10 g/L tryptone, 5 g/L yeast extract, 5 g/L NaCl, 7.63 g/L K_2_HPO4, 5 g/L Na_2_HPO4), supplemented with 50 μg/mL kanamycin, 100 μg/mL ampicillin, and 2 mM ammonium ferric citrate. When the OD600 reached 0.6, 0.5% (w/v) glucose and 2 mM cysteine were added. The culture was then bubbled with argon for 1 h to make it anaerobic. Once anaerobic, the protein production was induced with 0.5 mM IPTG and left for protein production overnight on the bench (20–25 °C) with stirring. Cells were then harvested under anaerobic conditions by centrifugation (6500 rpm, 30 min, 4 °C) and stored at −80 °C until the protein isolation and purification were carried out. The cells were resuspended in 100 mM Tris-HCl buffer, 150 mM NaCl, pH 8.0, and disrupted by sonication for 15 min (30% amplitude, cycles of 2 s on 10 s off). Following centrifugation, the protein was purified by a StrepTagII affinity column under strictly anaerobic conditions.

### Preparation of S75, D127, and S208 Apo-*Ca*HydA1 Truncated Proteins

*Ca*HydA1 truncation positions were identified by mapping the domains on InterPro (https://www.ebi.ac.uk/interpro/). The selected truncations are found in flexible loops that connect the domains, as seen in models built with AlphaFold2. Truncated genes were amplified by PCR using the following primers (S75-for: CTTTAAGAAGGAGATATACATATGTCCGATGAAGTAAAAGAACGAATC; D127-for: CTTTAAGAAGGAGATATACATATGGATAAGGATGCTCTAGTTGATAATAG; S208-for: CTTTAAGAAGGAGATATACATATGTCCCATATAGAAAAAGTTCAAG; rev: AGTGGTGGTGGTGGTGGTGCTCGAGTTATTTTTCAAATTGAGGATGACTCC), cloned in a pET-21a vector using the NEBuilder HiFi assembly cloning kit (NEB), and verified by DNA sequencing. The plasmids were transformed into *E. coli* BL21­(DE3)­Δ*iscR* and the proteins were expressed and purified following the same method described for the WT protein.

### In Vitro Artificial Maturation

WT and truncated proteins were artificially maturated by following previously published protocols.^33,61^ In brief, 100–200 μM apo-enzymes were mixed with a 3-fold excess of synthetic [2Fe] cofactor in buffer Tris-HCl, pH 8. The mixture was left to react for 1 h (with occasional pipetting) at room temperature. Afterward, the excess cofactor was removed via desalting on a PD-10 column.

### Protein Structure Prediction and Ferredoxin Docking

Models for *CaHyd*A1 and its truncated variants were generated using the Alphafold2 module within the Phenix software suite.^62,63^ FeS clusters were added to the resulting models using Alphafill (https://Alphafill.eu).^64^ The fit of the inserted clusters was optimized via the Alphafill web server to have TCS (transplant clash score) values below 0.29, which is considered a high confidence fit. Protein–protein docking of the natural redox partner *Ca*Fd was studied by ZDOCK.^65^

### Solution Activity Assays with Artificial and Physiological Redox Partners

H_2_ oxidation and H_2_ evolution were assayed by applying previously described methods using MV or *C. acetobutylicum* ferredoxin CAC0303 (*Ca*Fd) as electron mediator.^17^ All rates were measured at 37 °C in 100 mM Tris-HCl, 150 mM NaCl, pH 8. For the H_2_ evolution assay, H_2_ was quantified by gas chromatography (Agilent 7820A, purged packed inlet, Supelco Carboxen-1010 column, thermal conductivity detector, argon carrier gas) using either 10 mM reduced MV or 30 μM reduced *Ca*Fd as an electron donor. H_2_ oxidation assay was performed by UV–vis spectroscopy in a H_2_-saturated buffer containing either 10 mM oxidized MV or 30 μM oxidized *Ca*Fd as electron acceptor. An ε_604_ = 13.6 mM^–1^ cm^–1^ was used for reduced MV^20^ and ε_390_ = 32.6 mM^–1^ cm^–1^ was used for oxidized *Ca*Fd.^19^

### EPR Measurements

Continuous wave electron paramagnetic resonance (CW-EPR) data were collected at the Centre for Advanced ESR (CAESR) in the Department of Chemistry, University of Oxford. The X-band CW-EPR spectrometer was a Bruker EMXmicro with a Premium bridge and a 0.6 T electromagnet. The Bruker resonator was an ER4123 SHQE-W1 with an Oxford Instruments ESR900 cryostat, flowing helium cryogen set with an Oxford Instruments ITC503S temperature controller. Simulations of CW-EPR spectra utilized the complete spectrometer acquisition parameter values and nonsaturated spectra as inputs to EasySpin routines^66^ available in the Matlab (The Mathworks, Natick, NJ) scripting environment. General acquisition parameters are as follows: H_ox_ state signals were acquired at 25 K and 50–100 μW microwave power and 0.3 mT field modulation. Signals of the H_ox_-CO state were acquired at 60 K and 100 μW microwave power and 0.5 mT field modulation. Microwave frequencies were, for example, 9.4056, 9.4004, 9.3879, and 9.3883 GHz for H_ox_ WT, S75, D127, and S208, respectively. EPR samples were prepared with 150–200 μL of a 200 μM enzyme in 25 mM Tris-HCl, 30 mM KCl, pH 8. The X-band EPR tubes were sealed anaerobically and flash frozen, and stored in liquid nitrogen.

### FTIR Spectroscopy

FTIR spectra were measured in a commercial IR transmission cell (PIKE) on 5 μL of ≈1 mM samples (in 20 mM Tris-HCl, 30 mM NaCl, pH 8) sandwiched between two CaF_2_ windows (31.8 mm × 1.5 mm, Crystran) separated with a 25 μm Teflon spacer (PIKE Technologies). Spectra were measured at room temperature inside an anaerobic dry glovebox (Glove Box Technology Ltd., UK, O_2_ < 2 ppm, < 85 °C dew point) on a Bruker Vertex 80 FTIR spectrometer equipped with a nitrogen-cooled Bruker mercury cadmium telluride (MCT) detector. Spectra are an average of 512 scans with a resolution of 2 cm^–1^, and were collected in the double-sided, forward–backward mode with an aperture setting of 0.5 mm and a scan velocity of 20 kHz. Spectra were recorded using OPUS software, and data were processed using home-written routines in MATLAB. H_ox_-CO samples were prepared by flushing the sample for 20 min with carbon monoxide. To obtain the reduced states, the samples in 20 mM Tris-HCl, 30 mM NaCl, pH 8 buffer containing 10 mM sodium dithionite, were flushed with H_2_ for 1 h.

### Protein Film Electrochemistry

Electrochemical experiments were carried out in an electrochemical cell placed inside an anaerobic N_2_-filled glovebox (Belle Technologies, O_2_ < 3 ppm) using an Autolab potentiostat (PGSTAT128N) controlled by Nova software. H_2_ gas was flowed using mass flow controllers (Sierra Instruments). The PGE working electrode (area of 0.03 cm^2^) was rotated at a constant speed of 2000 rpm. A Pt wire was used as a counter electrode, and a saturated calomel electrode (SCE, PALMSENS BV) was used as a reference. The SCE reference electrode was kept in a separate side-compartment containing 0.1 M NaCl, which was connected to the main electrochemical cell compartment via a Luggin capillary. Potentials have been converted to volts vs the standard hydrogen electrode (SHE) using the correction *E*_SHE_ = *E*_SCE_ + 0.241 V at 25 °C.^67^ Each protein film was prepared under strictly anaerobic conditions inside a glovebox (Belle Technologies, O_2_ < 3 ppm) by first polishing the PGE electrode with sandpaper (P400, 3M), thoroughly rinsing it with ultrapure water and then adsorbing the enzyme (4 μL of ≈6 μM in 10 mM MES pH 5.8 buffer) by pipetting it directly onto the PGE surface and leaving it to adsorb for 5 min. Unbound protein was removed by rinsing the electrode with ultrapure water before inserting it into the electrochemical cell. Cyclic voltammograms were measured at 25 °C, 100% H_2_ (gas flow rate through the electrochemical cell headspace: 1 L/min), and in a mixed buffer (15 mM MES, HEPES, TAPS, CHES, sodium acetate, and 100 mM NaCl, adjusted to the desired pH).

## Supplementary Material



## Notes

Experimental procedures, biochemical characterization, electrochemical and spectroscopic data, and tables summarizing the data for the WT and truncated are available in the Supporting Information (PDF)
