# Supplementary material for: Engineering the Electron Relay in [FeFe]-Hydrogenase Enhances Electrocatalytic H2 Evolution
Source: ACS Catal. 2025 Nov 5;15(22):19216–26. doi: 10.1021/acscatal.5c03665 (PMC12645427; doi:10.1021/acscatal.5c03665)
Supplement: Supplementary file 1 [file cs5c03665_si_001.pdf]

Supporting Information

Engineering the electron relay in [FeFe]-hydrogenase enhances electrocatalytic H_2 evolution

Tin Pou Lai,[1] William K. Myers,[1] Stephen B. Carr,[1,2] Miguel A. Ramirez,[1] Kylie A. Vincent, [*1] Simone Morra[*3] and Patricia Rodríguez-Maciá [*1,4]

[1]University of Oxford, Department of Chemistry, Inorganic Chemistry Laboratory, South Parks Road, Oxford, OX1 3QR, UK.

[2]Research Complex at Harwell, Rutherford Appleton Laboratory, Harwell Campus, Didcot, U.K.

[3]University of Nottingham, Faculty of Engineering, Coates Building, University Park, Nottingham, NG7 2RD, UK.

[4]School of Chemistry and Leicester Institute for Structural and Chemical Biology, University of Leicester, University Road, Leicester, LE1 7RH, UK.

* kylie.vincent@chem.ox.ac.uk; simone.morra@nottingham.ac.uk; prm28@leicester.ac.uk.

Table of Content

Overexpression and purification of apo-*Ca*HydA1 and its truncated variants 3
Figure S1 4
Figure S2 4
Figure S3 5
Figure S4 5
Figure S5 6
Figure S6 7
Figure S7 7
Figure S8 8
Figure S9 9
Figure S10 10
Figure S11 10
Figure S12 11
Figure S13 12
Figure S14 12
Figure S15 13
Figure S16. 14
Figure S17 15
Figure S18 16
Figure S19 17
Table S1 17
Table S2 17
Table S3A 18
Table S3B 18
Table S3C 18
Table S4 19
Table S5 19
Table S6 19
Table S7 20
Table S8 20
Supplementary References 21

Overexpression and purification of apo-*Ca*HydA1 and its truncated variants.

The S75 variant was truncated at residue serine 75, deleting only the [2Fe-2S] cluster in the F-domain, has a molecular weight of 57.5 kDa and holds three additional FeS clusters. The M2-type *Dd*HydAB-like truncated protein D127 begins at residue aspartate 127 (deleting the [2Fe-2S] cluster and the His-ligated [4Fe-4S] cluster) and retains two [4Fe-4S] clusters (all Cys-ligated) in the F-domain plus the [4Fe-4S] cluster of the H-cluster. D127 has a molecular weight of 51.4 kDa. The M1 type *Cr*HydA1-like truncated protein S208 was truncated at residue serine 208 and contains only the H-cluster. S208 has a molecular weight of 42.8 kDa. Compared to the *'in vivo'* maturation system (i.e., preparations by native maturases), yielding only 2 mg of holo-*Ca*HydA1 from 1 L of culture, the semi synthetic overexpression method for apo-*Ca*HydA1 production, the yield is doubled (5 ± 1 mg per 1 L culture, Figure S1 and S2). Furthermore, the yield is increased to 20 ± 4 mg/ L for the D127 truncated protein and to 28 ± 5 mg/ L for the S208 truncated protein. However, S75 suffers from low expression levels, producing only 3 ± 1 mg/ L, which could imply that the [2Fe-2S] cluster containing N-terminus domain affects the protein folding and is crucial for stability (see Table S1 below for yields). The Fe contents of the WT *Ca*HydA1 and truncated proteins are confirmed by ICP-MS (Table S2) and the FeS clusters are studied via UV-VIS spectroscopy (Figure S3).

Supplementary Figures

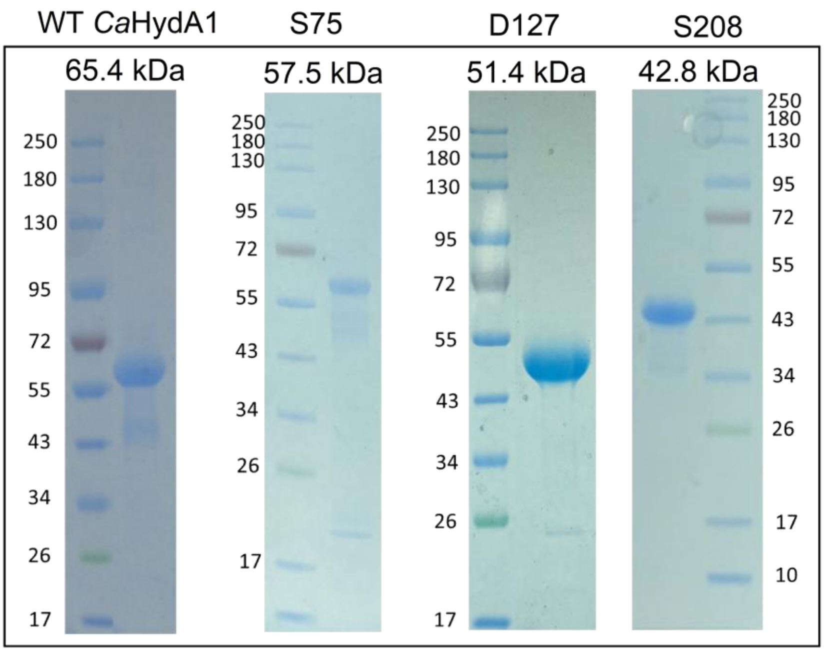

Figure S1. SDS-PAGE of apo-*Ca*HydA1 [FeFe] hydrogenases found at 65.4 kDa for WT, 57.5 kDa for S75, 51.4 kDa for D127 and 42.8 kDa for S208.

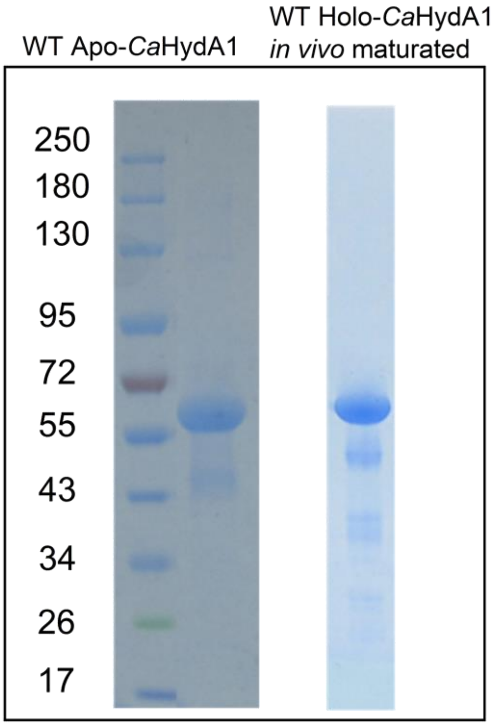

Figure S2. SDS-PAGE of WT apo-*Ca*HydA1 [FeFe] hydrogenase after single StrepTagII affinity column found at 65 kDa, WT holo-*Ca*HydA1 [FeFe] hydrogenase in vivo maturated after StrepTagII and size exclusion column.

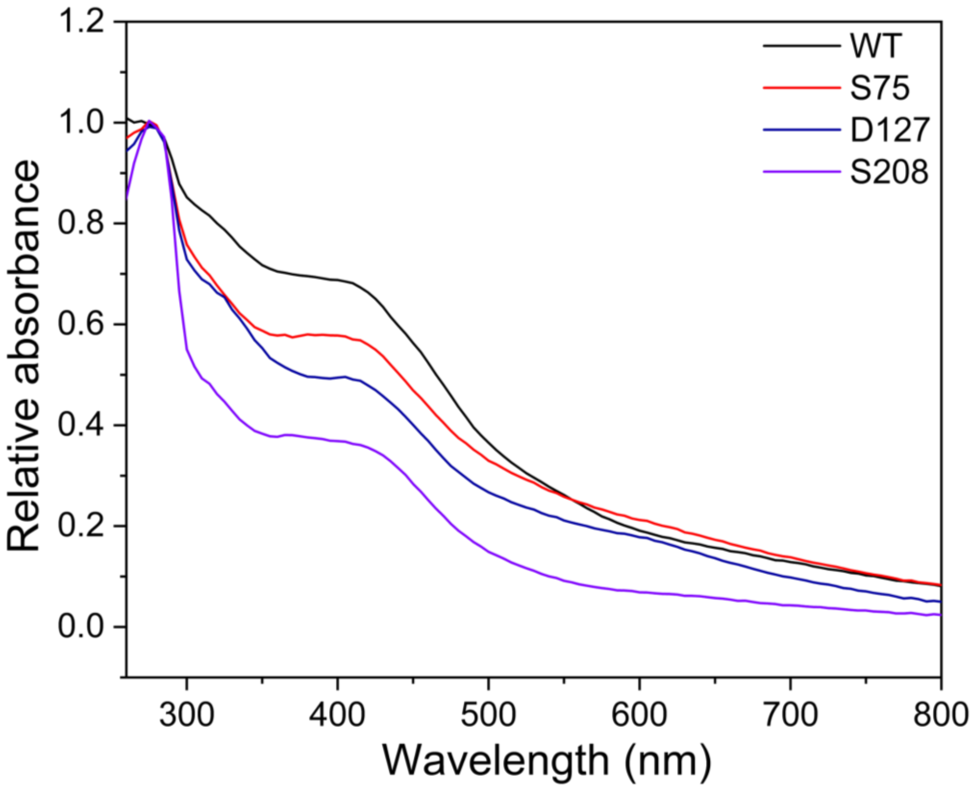

Figure S3. UV-VIS spectra of apo-*Ca*HydA1 WT and truncated S75, D127 and S208 variants. A band at around 410 nm corresponding to FeS clusters.

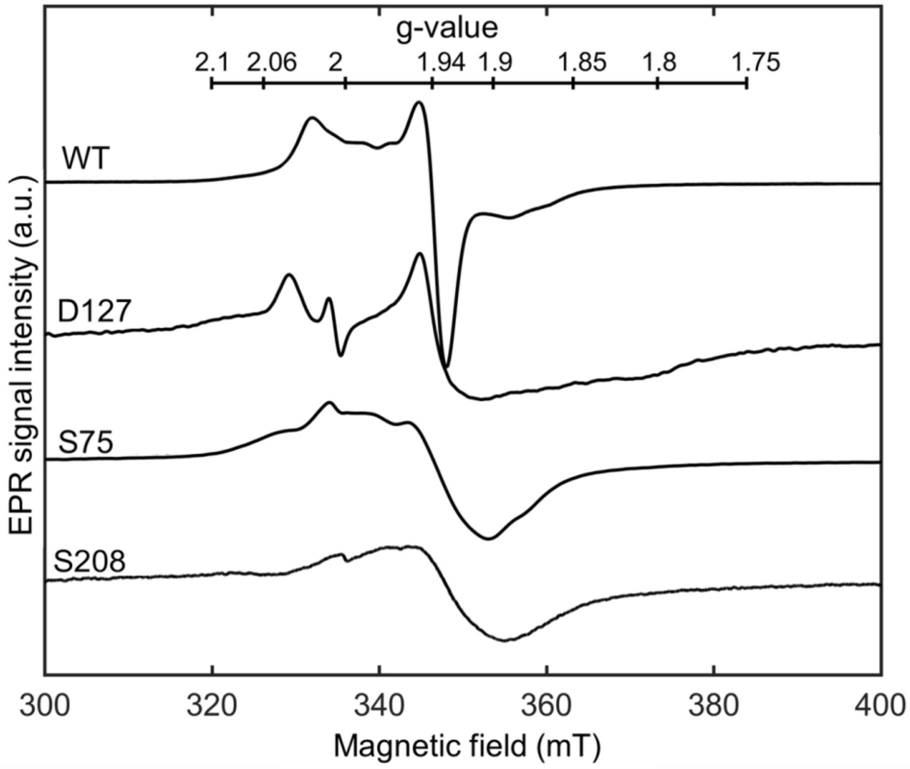

Figure S4. Comparison of EPR spectra (9.4 GHz, T=10 K) of 200 µM apo-*Ca*HydA1 WT and truncated S75, D127 and S208 variants reduced with 10 mM NaDT in 25 mM Tris-HCl, 30 mM KCl, pH=8 collected at 10 K and non-saturating conditions. The g-value bar is indicated on top of the spectra.

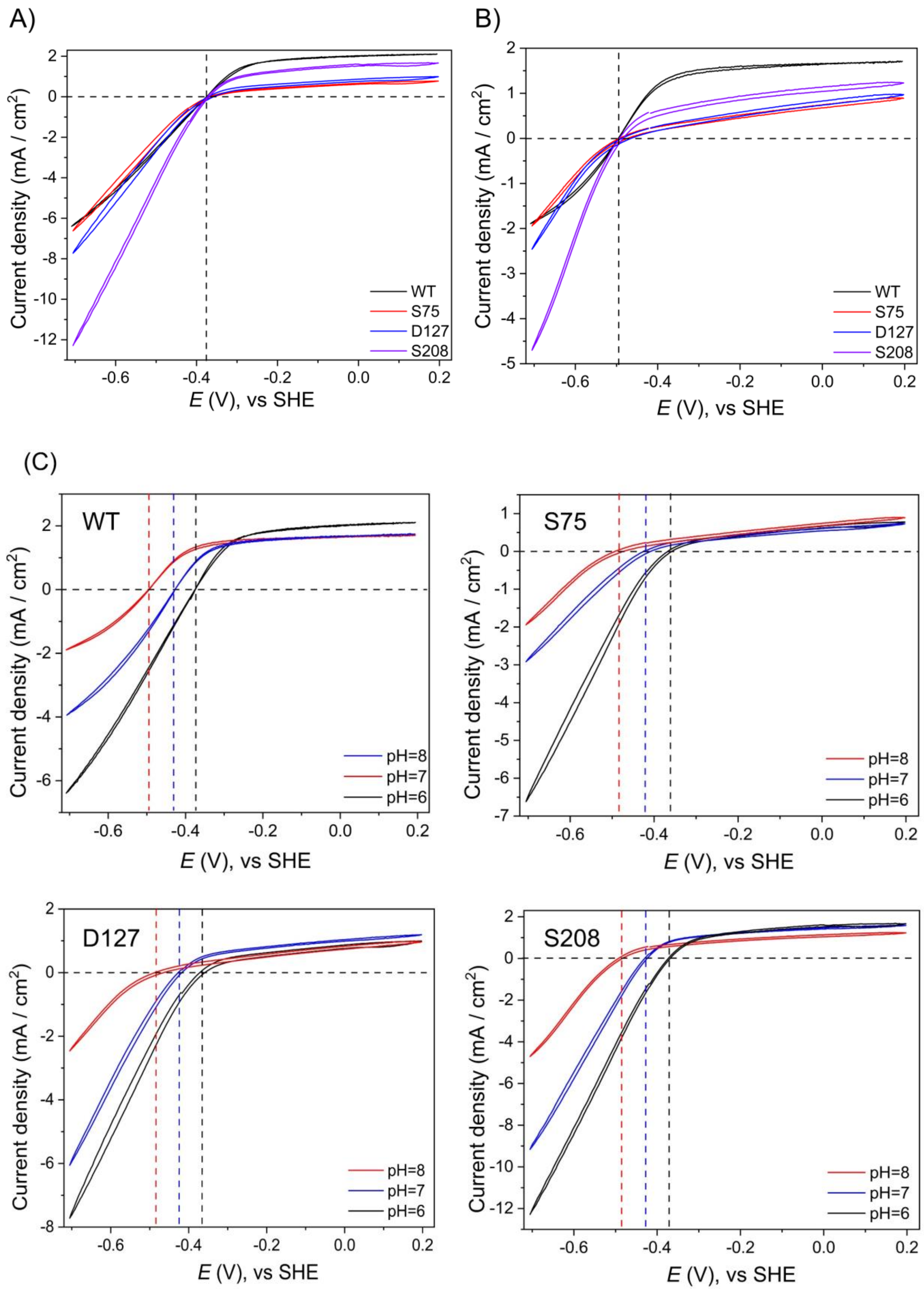

Figure S5. Comparison of the electrochemical behaviour at different pH values for WT *Ca*HydA1 and truncated proteins. A) Cyclic voltammograms of proteins absorbed onto a rotating pyrolytic graphite electrode at pH 6; B) Cyclic voltammograms of *Ca*HydA proteins absorbed onto a rotating pyrolytic graphite electrode at pH 8. C) Comparison of the electrochemical behaviour at three different pH values for each of WT *Ca*HydA1 and

truncated proteins. All measured in buffer mix at the desired pH, 25 °C, 2000 rpm rotation rate, 20 mV/s scan rate, and under 1 atm of H_2 (1000 mL/ min). The horizontal dashed line represents the zero current, and the vertical dashed line indicates the thermodynamic potential of the $2H^+/H_2$ couple at the given pH.

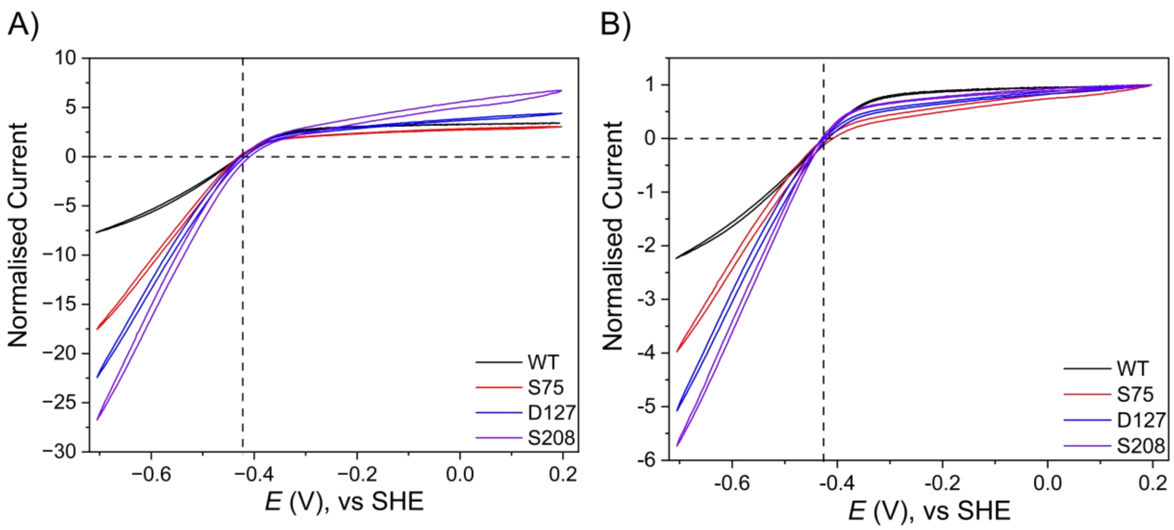

Figure S6. Normalized CVs of WT *Ca*HydA1and truncated proteins absorbed onto a pyrolytic graphite electrode at pH=7. All measured in buffer mix, 25 °C, 2000 rpm rotation rate, 20 mV/s scan rate, and under 1 atm of H_2. A) Normalisation at -400 mV. B) Normalisation using the highest oxidation current. The horizontal dashed line represents the zero current, and the vertical dashed line indicates the thermodynamic potential of the $2H^+/H_2$ couple at the given pH.

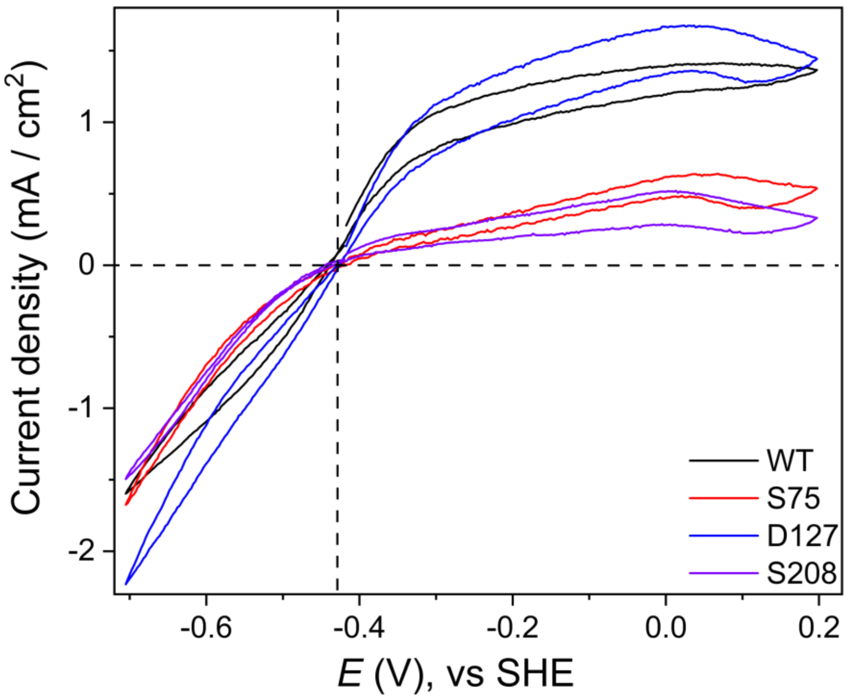

Figure S7. CV of WT *Ca*HydA1 and truncated proteins absorbed onto a pyrolytic graphite electrode at pH 7 measured at a slow scan rate. All measured in buffer mix, 25 °C, 2000 rpm rotation rate, 5 mV/s scan rate, and

under 1 atm of H_2. The horizontal dashed line represents the zero current, and the vertical dashed line indicates the thermodynamic potential of the $2H^+/H_2$ couple at the given pH.

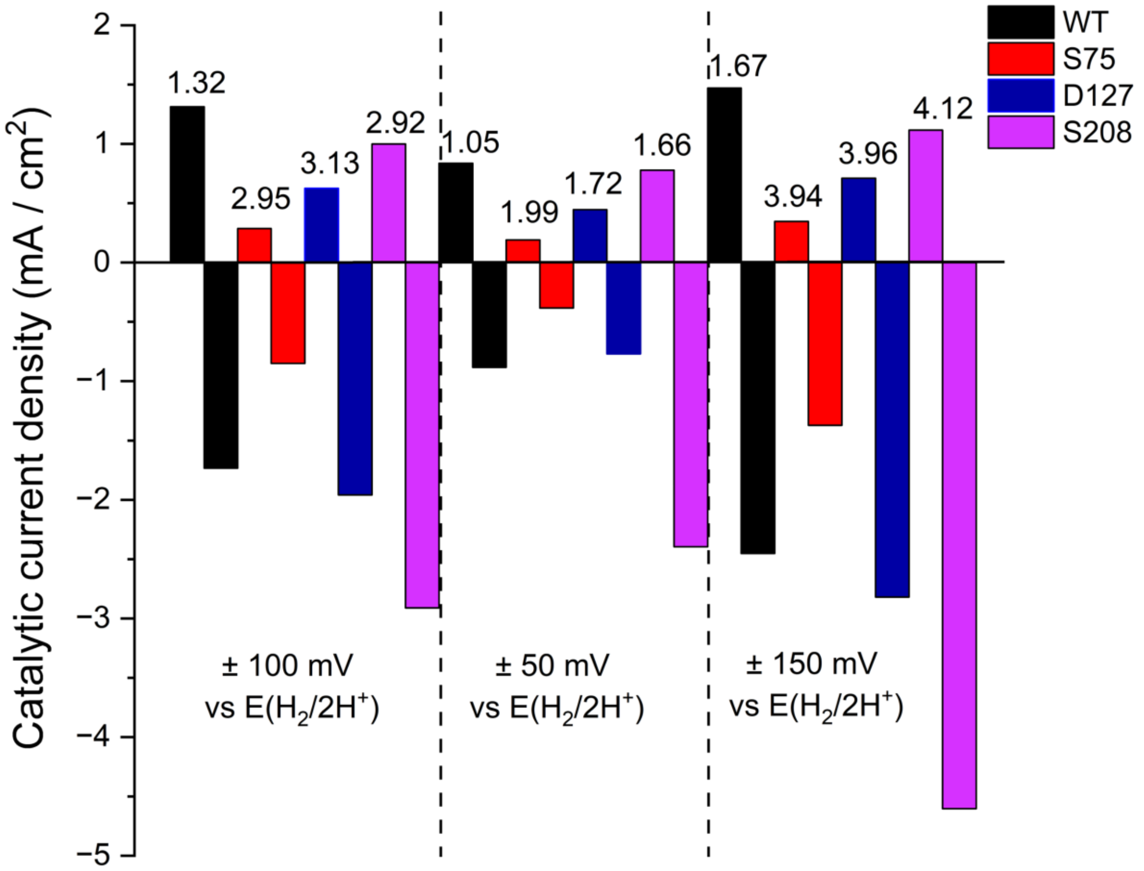

Figure S8. Determination of the catalytic bias at three different overpotentials for WT, truncated *Ca*HydA1 [FeFe] hydrogenases from protein film electrochemistry at pH 7 (by calculating the ratio of catalytic current density on the reduction direction / oxidation direction, values are indicated the top of at each column) at ± 100 mV vs E($2H^+/H_2$), ± 50 mV vs E($2H^+/H_2$) and ± 150 mV vs E($2H^+/H_2$). WT and S75, D127 and S208 variants are highlighted in black, red, blue and purple respectively.

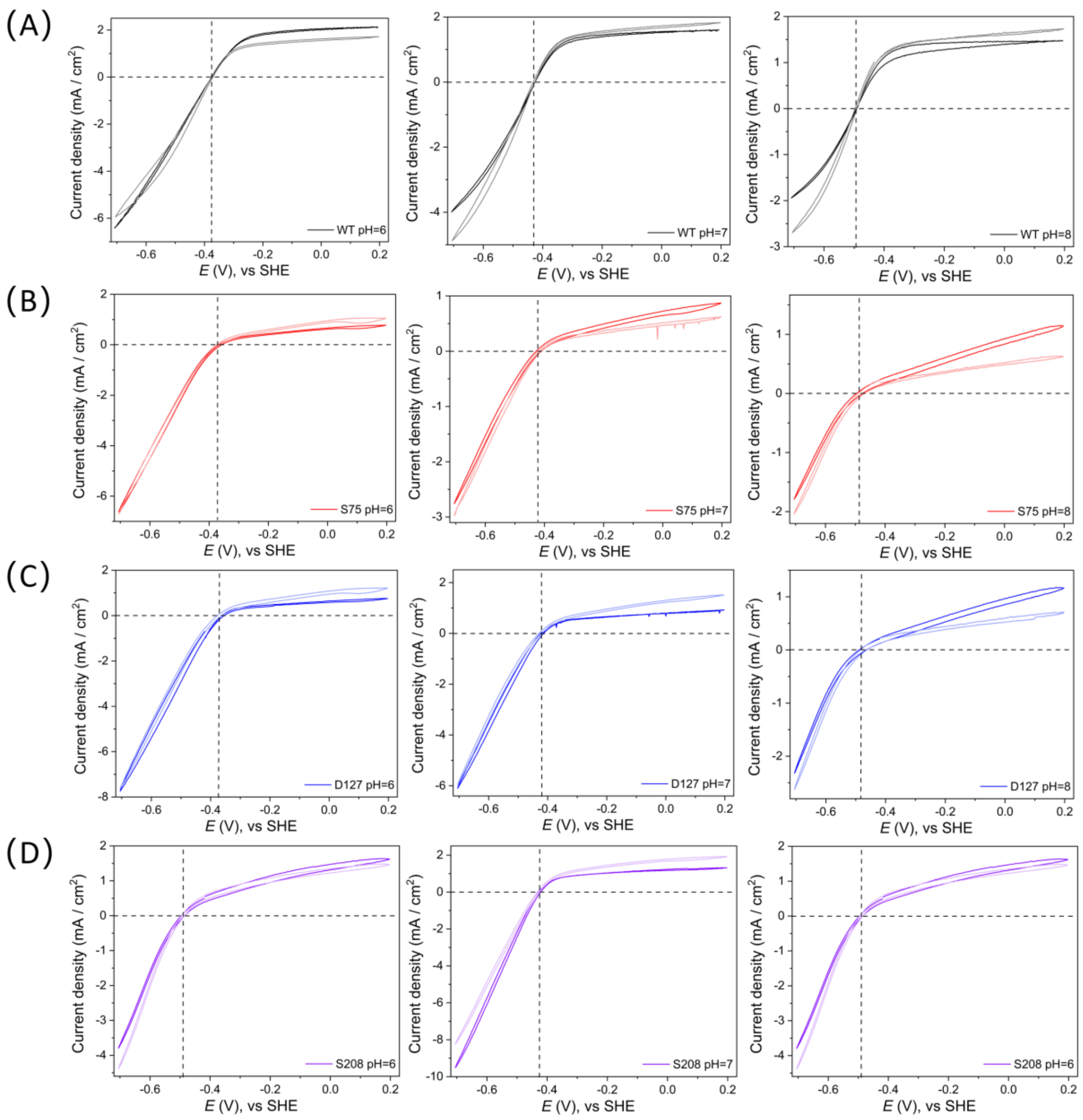

Figure S9. Duplicates of film preparations for every enzyme at different pH values . CVs of A) WT, B) S75, C) D127, and D) S208 *Ca*HydA1 [FeFe]-hydrogenases adsorbed onto a pyrolytic graphite electrode measured at pH 6, 7 and 8, at room temperature, constant flow of 100% H_2 (1 L/ min), 2000 rpm and 20 mV/s scan rate. The horizontal dashed line represents the zero current, and the vertical dashed line indicates the thermodynamic potential for the $2H^+/H_2$ couple at the given pH.

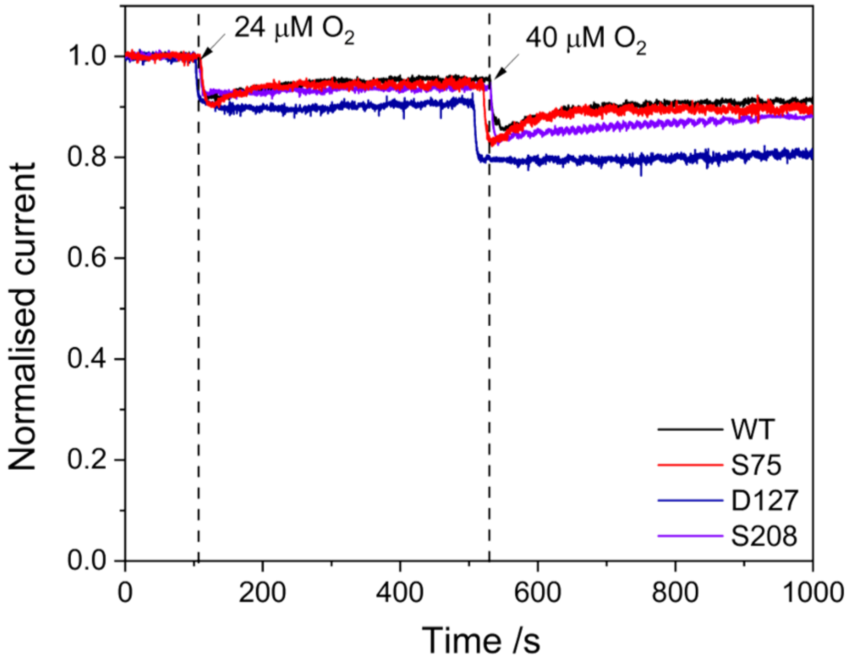

Figure S10. Normalized chronoamperogram showing aerobic inactivation of WT *Ca*HydA1 and its truncated variants. The enzymes were absorbed onto a rotating pyrolytic graphite electrode and poised at +40 mV vs SHE under a constant flow of 100% H_2 to flush out the O_2. The H_2 oxidation current was monitored vs time following the injection of small amounts of O_2 in the electrochemical cell. The solid lines are the film loss corrected data by subtracting the experimental data to the fitted data extrapolated from the anaerobic part of the data (recorded in first 100 seconds) by fitting to an exponential function.[1] The data were normalized in the range of [1,0] using OriginPro software by setting the initial current at t = 0 to 1 and the baseline (zero current) to 0.

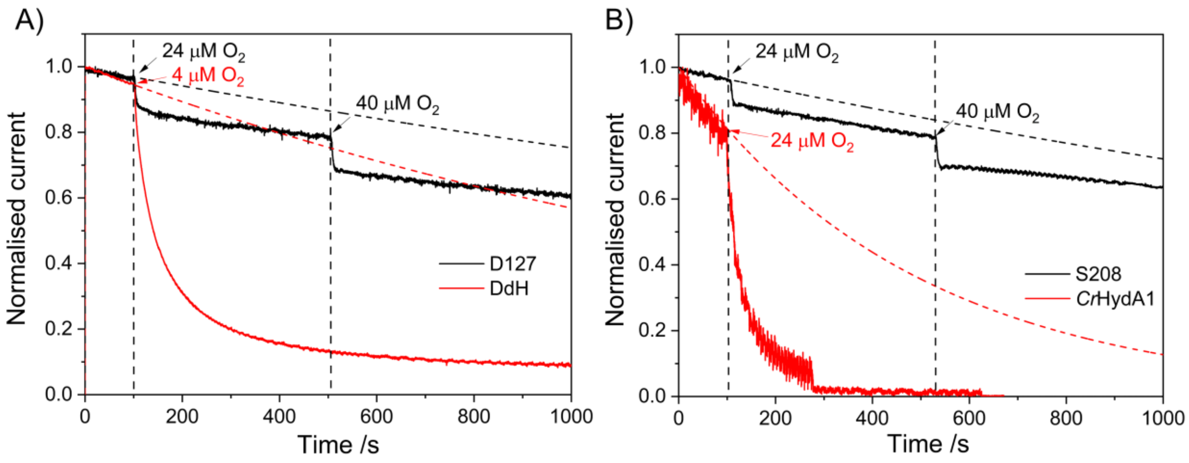

Figure S11. Aerobic inactivation comparison between M2-type like D127 and the enzyme *Dd*HydAB and M1-type like S208 and *Cr*HydA1. Enzymes were absorbed onto a PGE and the potential was poised at +40 mV vs SHE under 100 % H_2 atmosphere. The H_2 oxidation current was monitored vs time following the injection of small amounts of air-saturated buffer into the electrochemical cell. The solid lines are the experimental data and dotted lines are the projected current baselines accounting for film loss,[1] extrapolated from the anaerobic part of the data (recorded in first 100 seconds) by fitting to an exponential function. Vertical dash lines indicate the points where O_2 was added to the electrochemical cell. The data were normalized in the range of [1,0] using OriginPro software by setting the initial current at t = 0 to 1 and the baseline (zero current) to 0.

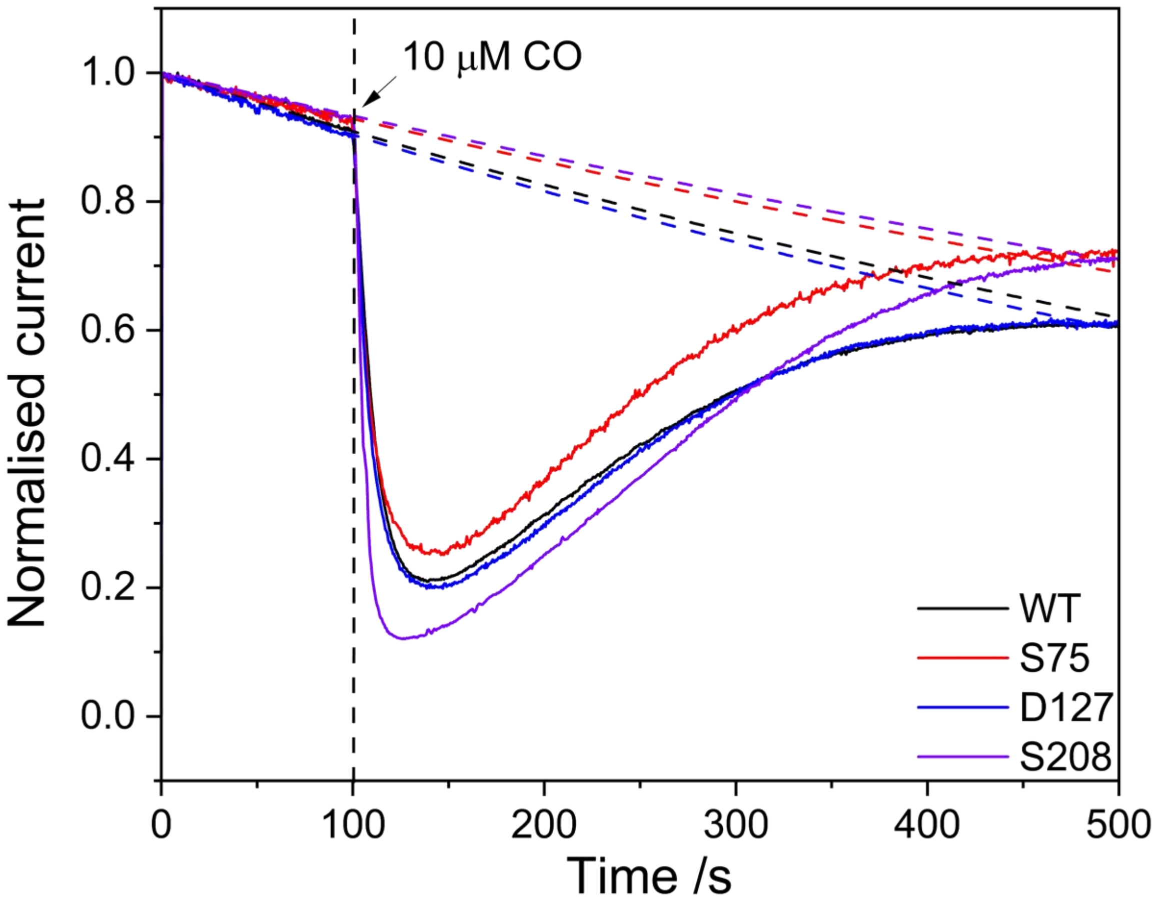

Figure S12. Normalized chronoamperogram showing the CO inhibition of WT *Ca*HydA1 and its truncated variants. The enzymes were absorbed onto the rotating pyrolytic graphite electrode and poised at -160 mV vs SHE under 100 % H_2 atmosphere. The H_2 oxidation current was monitored vs time following the injection of small amounts of CO in the electrochemical cell. The solid lines are the experimental data and dotted lines are the projected current baselines accounting for film loss.[1] Vertical dash lines indicate the points where O_2 was added to the electrochemical cell. The data were normalized in the range of [1,0] using OriginPro software by setting the initial current at t = 0 to 1 and the baseline (zero current) to 0.

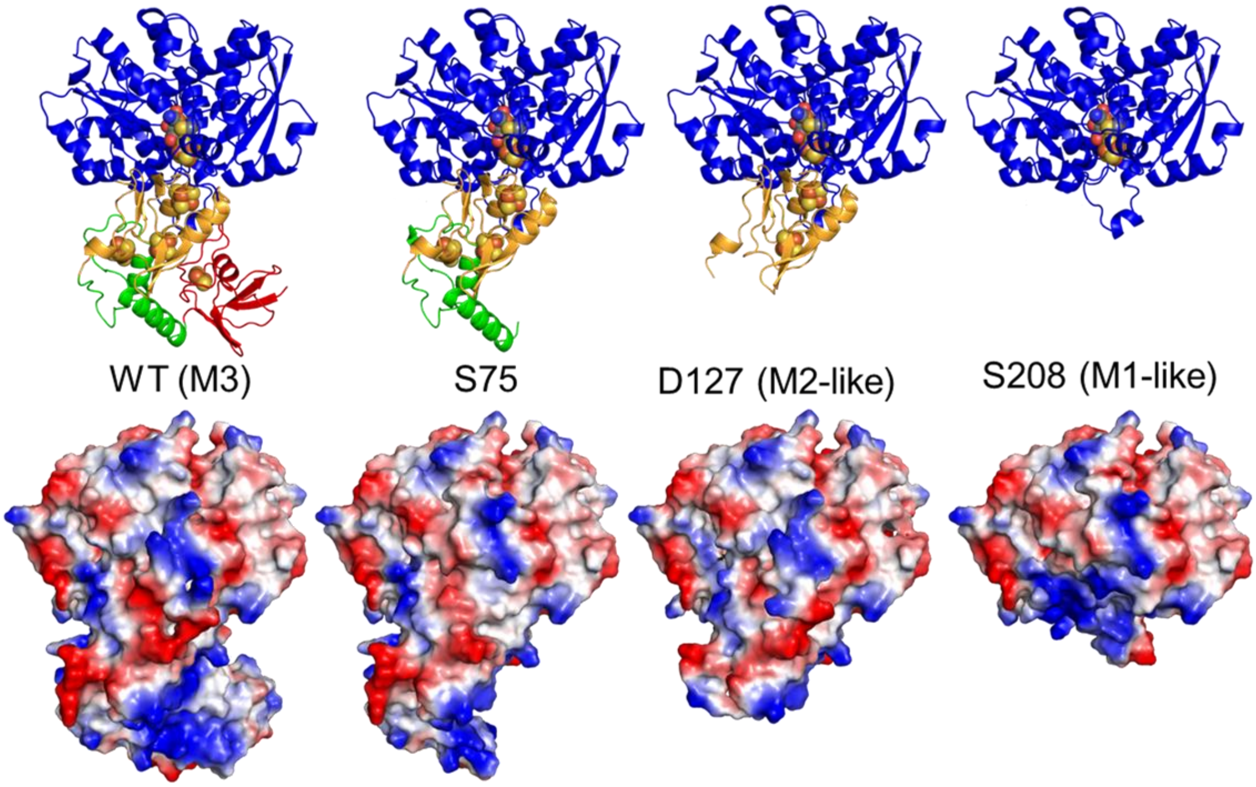

Figure S13. Electrostatic surface prediction of *Ca*HydA1 and truncated enzymes. Vacuum electrostatics were computed in PyMOL. Blue patches represent positive areas, red patches represent negative areas.

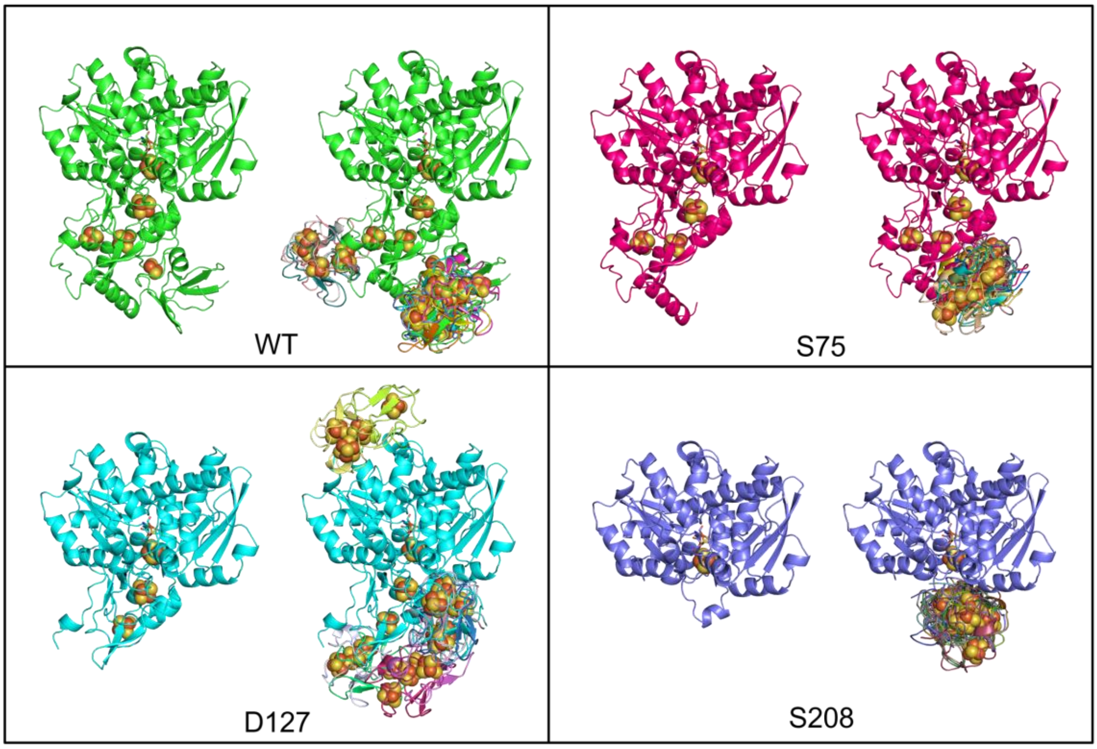

Figure S14. Top 10 docking predictions of *Ca*Fd binding to *Ca*HydA1 and its truncated variants based on AlphaFold2 models. Four sets of WT enzyme and truncated variants are displayed in four different colors. In

each set, each construct is shown on the left and the binding predictions of *Ca*Fd to the constructs are shown on the right.

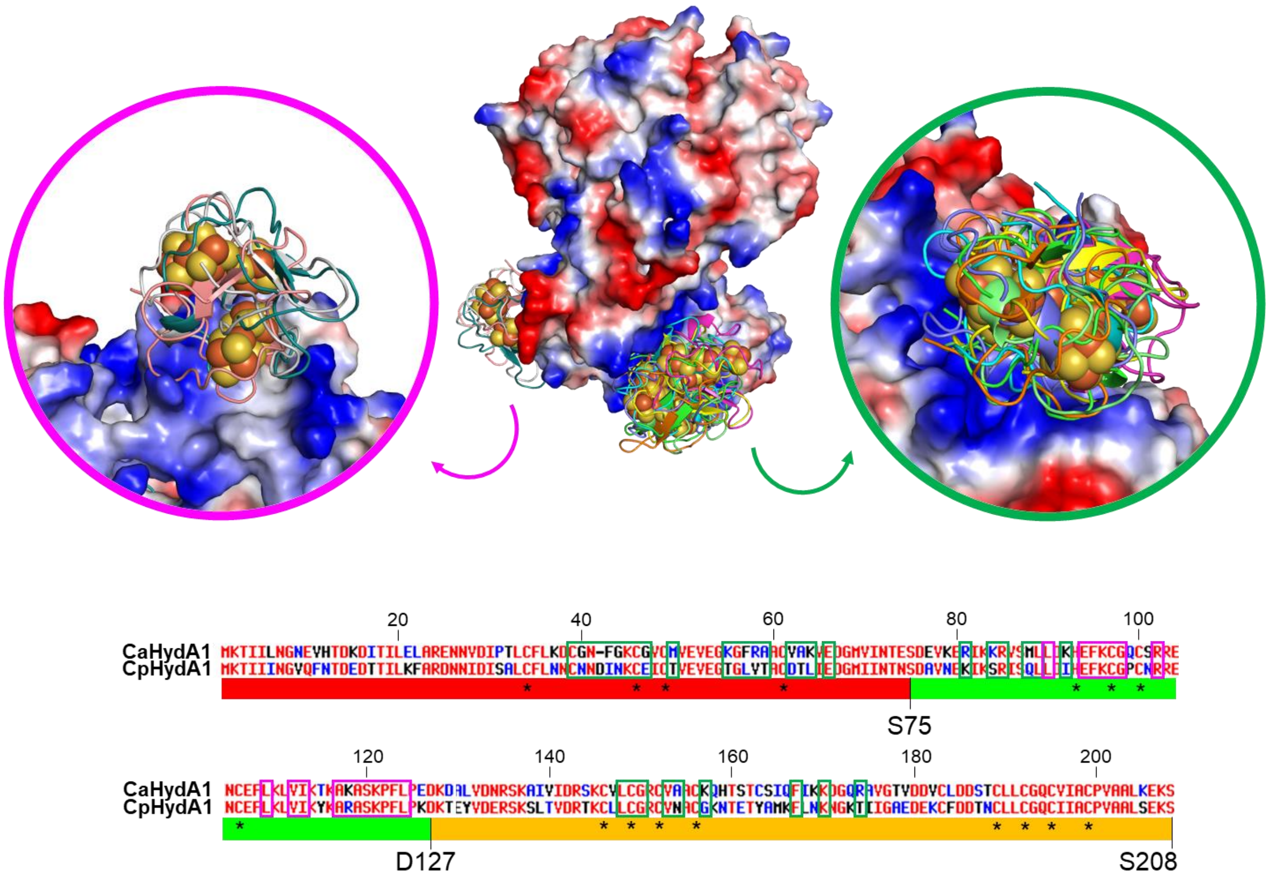

Figure S15. Docking predicts *Ca*Fd binding preferentially at the [2Fe-2S] domain of *Ca*HydA1. Within the top 10 poses, 7 predict binding at site 1 (green insert), while only 3 predict binding at site 2 (magenta insert). Both predicted binding sites are lined in positively charged residues, with the most favoured (site 1) spanning across the [2Fe-2S] domain FS2 (residues 1-74) and the 2x[4Fe4S] domain FS4A/B (residues 127-207), while site 2 is found uniquely on the histidine-ligated [4Fe4S] domain FS4C (residues 75-126). Despite high overall sequence identity between *Ca*HydA1 and *Cp*HydA1 (70.5%), significant differences exist locally, particularly at surface residues that are predicted to form site 1 (green squares), suggesting that the natural redox partner may bind differentially due to these inherent electrostatic differences. Models and colour codes are the same as figures S9-S10. Within the sequence alignment, red residues are high consensus, blue and black residues are low consensus; asterisks denote cysteines/histidines that coordinate FeS clusters in the hydrogenase. Green squares highlight hydrogenase residues that form binding site 1 (most favoured), magenta squares highlight residues that form binding site 2 (less favoured).

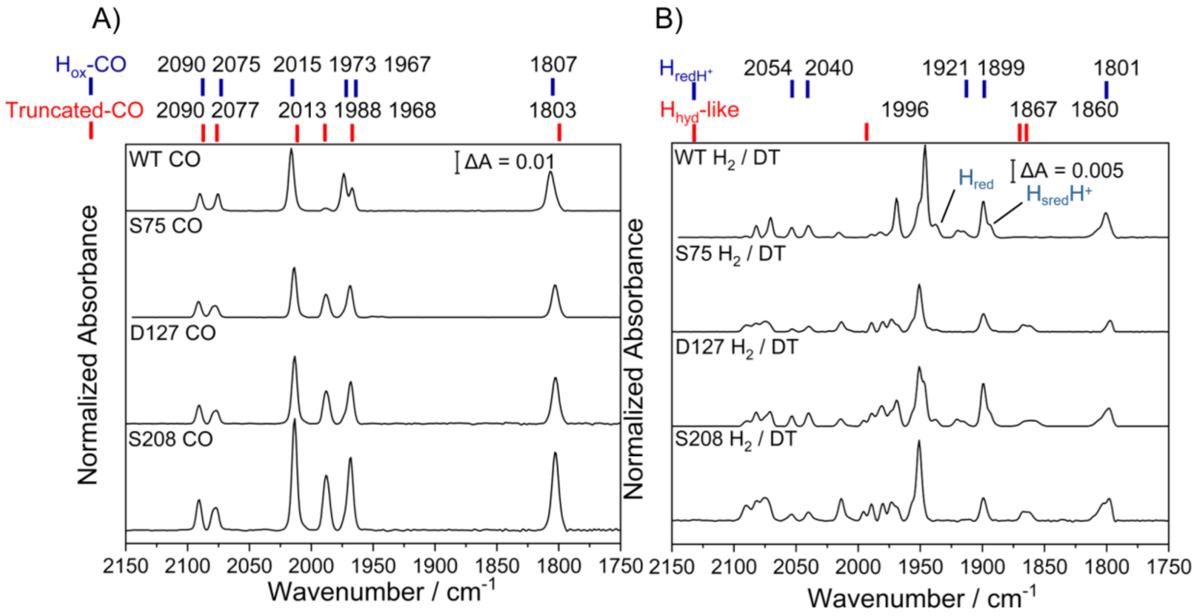

Figure S16. FTIR spectra of WT and truncated *Ca*HydA1 proteins at pH 8, inhibited with CO to form the H_{ox}-CO state (A), and reduced by addition of 10 mM sodium dithionite (NaDT) under H_2 atmosphere (B). The main observed states are highlighted in blue and red on top. Sample concentration is ≈ 1 – 2 mM, prepared in a N_2 glovebox, spectra are an average of 512 scans measured at RT and at 2 cm^{-1} resolution. The peaks marked with * correspond to the H_{ox}-CO state.

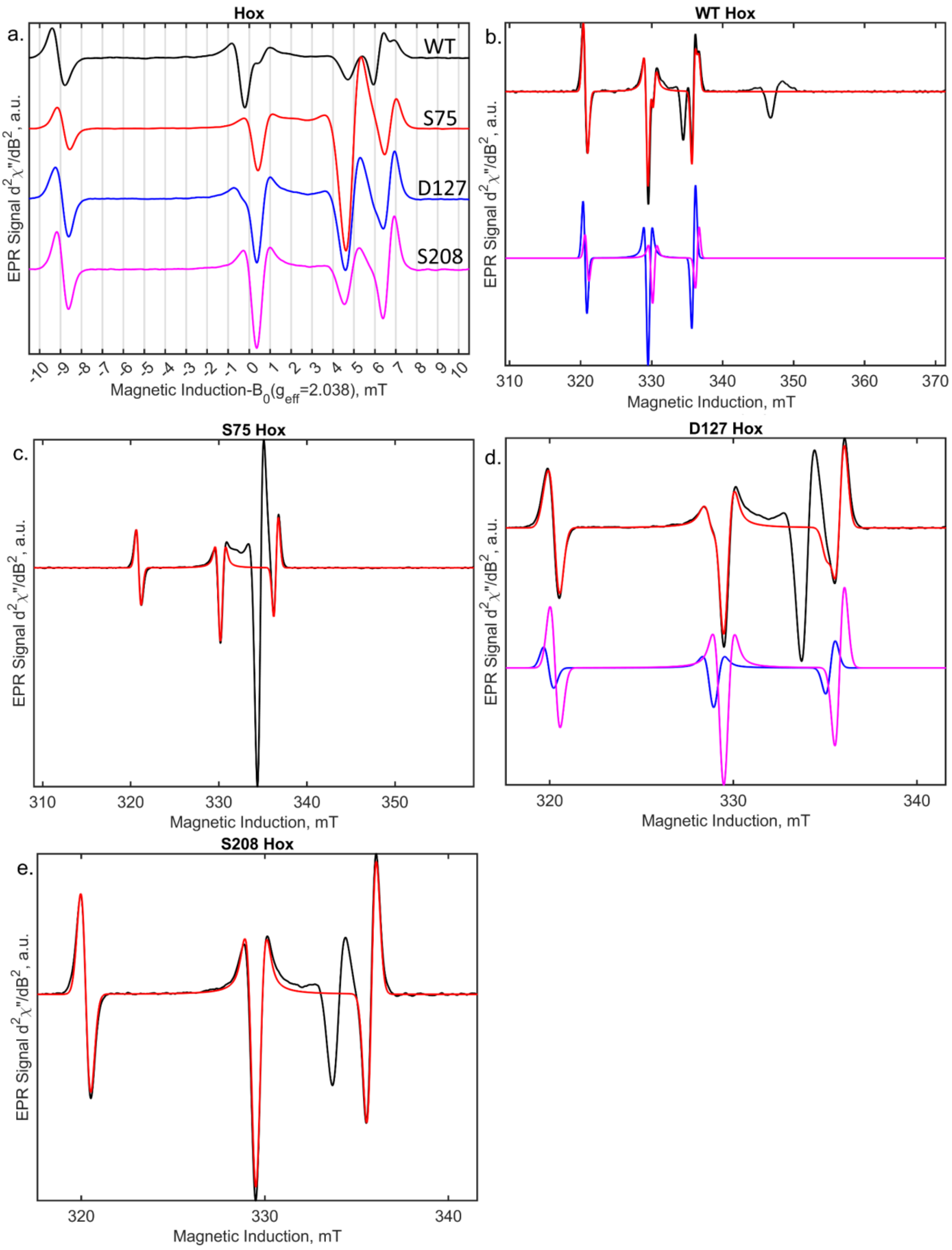

Figure S17. Simulations of the Hox state of numerical second derivatives via 0.5 mT pseudomodulation of CW-EPR spectra at X-band of 200 µM wild type (WT) and truncated S75, D127, and S208 proteins. Simulation values are included in Table S8. Panel a. shows all data as second derivatives, whilst panels b-e have corresponding total simulations in red and components in blue & violet. All samples contain fractional H_{ox}-CO states, while WT additionally contains a $[2Fe\text{-}2S]^{+}$ signal.

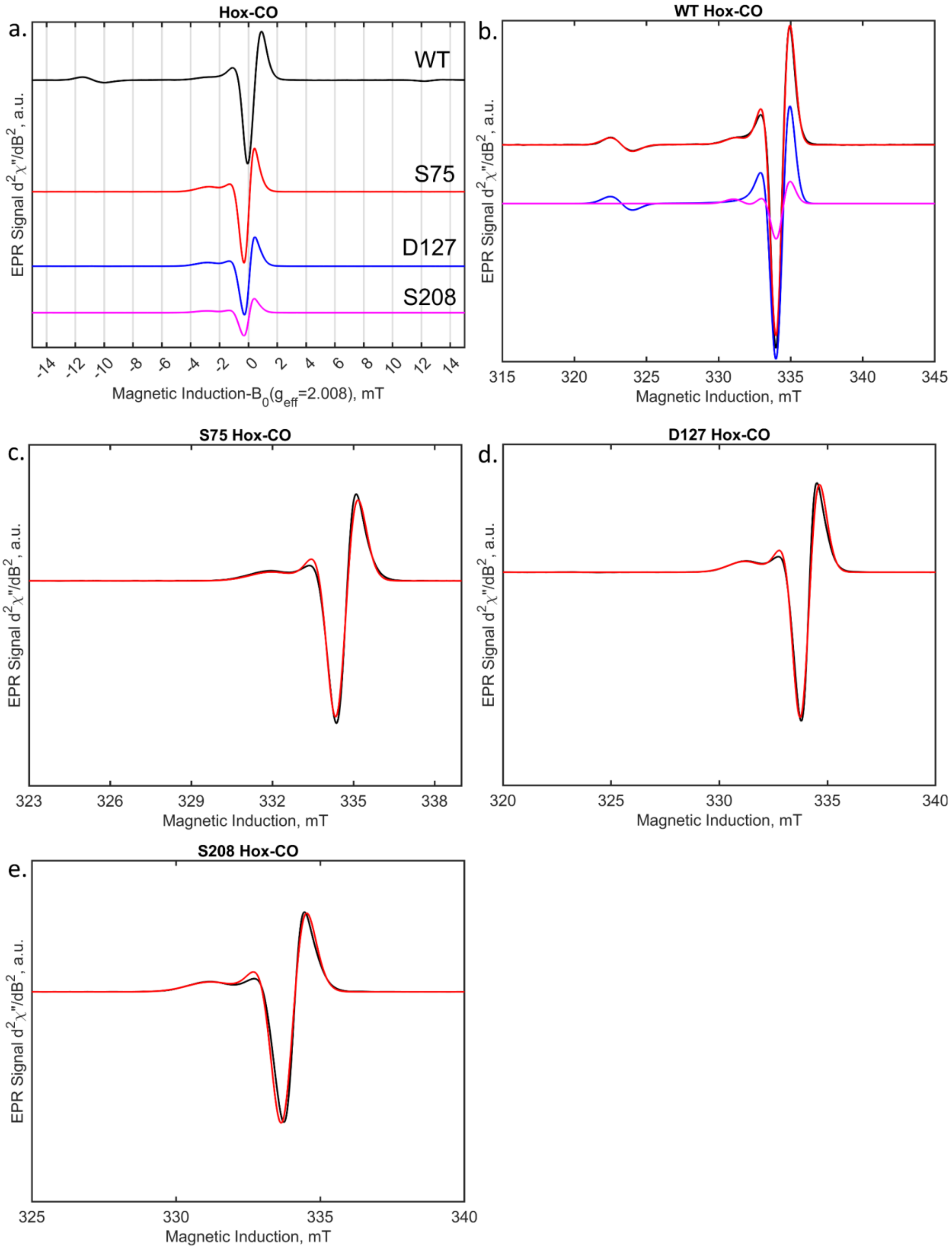

Figure S18. Simulations of the H_{ox}-CO state of numerical second derivatives via 0.5 mT pseudomodulation of CW-EPR spectra at X-band of 200 µM wild type (WT) and truncated S75, D127, and S208 variants. Simulation values are included in Table S8. Panel a. shows all data as second derivatives, whilst panels b-e have corresponding total simulations in red and components in blue & violet.

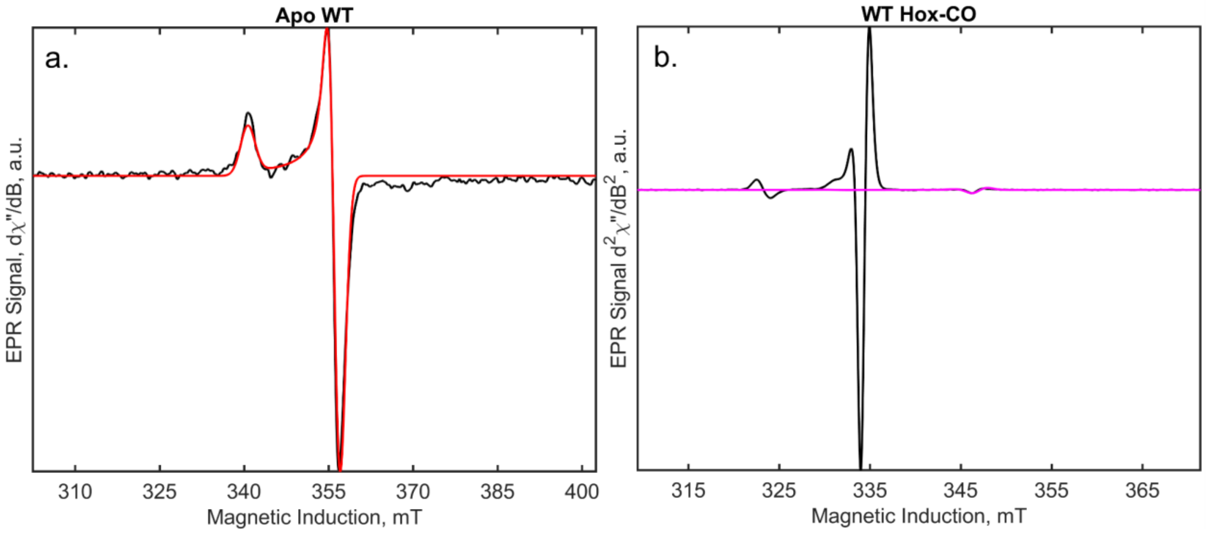

Figure S19. Simulation (red) of the [2Fe-2S]+ signal apo CaHydA1 [FeFe] hydrogenase (black), at 25 K panel a., and corresponding simulation of the [2Fe-2S]+ signal (magenta) in holo Hox-CO state (black), panel b., showing relative intensity to second Hox-CO component in WT protein. Simulation values are included in Table S8.

Supplementary Tables

Table S1. The production yield of the WT and truncated proteins (S75, D127 and S208)

	WT apo-*Ca*HydA1	S75	D127	S208
Yield /mg L^{-1} of culture	5 ± 1	3 ± 1	20 ± 4	28 ± 5

Table S2. Determination of the Fe content by ICP-MS and expected Fe contents of the holo-*Ca*HydA1 WT and truncated proteins (S75, D127 and S208).

	WT *Ca*HydA1	S75	D127	S208
Fe content observed	22.0 ± 1.4	18.5 ± 1.2	10.6 ± 0.5	6.7 ± 0.3
Fe content expected	20	18	14	6

Table S3A. Determination of the catalytic bias of WT, truncated *Ca*HydA1 [FeFe] hydrogenase from protein film electrochemistry at pH 7 and at +/-100 mV vs E(H_2/2H^+).

	WT *Ca*HydA1	S75	D127	S208
Catalytic current density -100 mV vs H_2/2H^+ potential (mA cm^{-2})	-1.733	-0.849	-1.958	-2.910
Catalytic current +100 mV vs H_2/2H^+ potential (mA cm^{-2})	+1.314	+0.288	+0.626	+1.001
Catalytic bias (H_2 production/ H_2 oxidation)	1.32	2.95	3.13	2.92

Table S3B. Determination of the catalytic bias of WT, truncated *Ca*HydA1 [FeFe] hydrogenase from protein film electrochemistry at pH 7 and at +/-50 mV vs E(H_2/2H^+).

	WT *Ca*HydA1	S75	D127	S208
Catalytic current density -50 mV vs H_2/2H^+ potential (mA cm^{-2})	-0.882	-0.382	-0.769	-1.295
Catalytic current density +50 mV vs H_2/2H^+ potential (mA cm^{-2})	0.838	0.192	0.446	0.779
Catalytic bias (H_2 production/ H_2 oxidation)	1.05	1.99	1.72	1.66

Table S3C. Determination of the catalytic bias of WT, truncated *Ca*HydA1 [FeFe] hydrogenase from protein film electrochemistry at pH 7 and at +/-150 mV vs *E*(H_2/2H^+).

	WT *Ca*HydA1	S75	D127	S208
Catalytic current density -150 mV vs H_2/2H^+ potential (mA cm^{-2})	-2.452	-1.371	-2.819	-4.602
Catalytic current density +150 mV vs H_2/2H^+ potential (mA cm^{-2})	1.471	0.348	0.712	1.117
Catalytic bias (H_2 production/ H_2 oxidation)	1.67	3.94	3.96	4.12

Table S4. Determination of the E_{swtich} for WT and truncated *Ca*HydA1 [FeFe] hydrogenase from protein film electrochemistry at pH 7, **5 mV/s scan rate** after calculating the minimum first derivative from Figure S6.

	WT *Ca*HydA1	S75	D127	S208
Minimum 1st derivative (10^{-5})	NA	-3.05	-6.47	-3.63
E_{swtich} / mV, vs SHE	NA	+78	+73	+65

Table S5. Solution activity assays of WT *Ca*HydA1 and truncated variants. Data shows the exact numbers, the activities relative to WT and bias between H_2 evolution and uptake.

	**H_2 uptake rate /s^{-1}**	**Relative to WT**	**H_2 evolution rate /s^{-1}**	**Relative to WT**	**Bias (evol/upt)**
**WT**	2744.0 ± 301.7		1640.9 ± 111.0		59.8 ± 7.7%
**S75**	243.7 ± 32.4	8.9%	149.7 ± 18.0	9.1%	61.4 ± 11.0%
**D127**	364.9 ± 23.7	13.3%	267.4 ± 51.5	16.3%	73.3 ± 14.9 %
**S208**	823.6 ± 111.1	30.0%	396.6 ± 48.2	24.2%	48.2 ± 8.7%

Table S6. Solution activity assays of WT *Ca*HydA1 and truncated variants using native redox partner *Ca*Fd. Data shows the exact numbers, the activities relative to WT and bias between H_2 evolution and uptake.

	**H_2 uptake rate /s^{-1}**	**Relative to WT**	**H_2 evolution rate /s^{-1}**	**Relative to WT**	**Bias (evol/upt)**
**WT**	281.4 ± 32.3		1055 ± 12		3.75
**S75**	0.2 ± 0.1	0.08%	2.2 ± 0.2	0.21%	9.61
**D127**	0.3 ± 0.1	0.11%	2.4 ± 0.4	0.22%	7.84
**S208**	0.8 ± 0.2	0.29%	4.8 ± 0.9	0.45%	5.80

Table S7. IR band summary of WT *Ca*HydA1 and its truncated variants.

CaHydA1	Redox state	FTIR signals (cm^{-1})
**WT *Ca*HydA1**	H_{ox}	2082, 2070, 1969, **1946**, 1801
	H_{ox}^{PDT}	2083, 2072, 1971, **1948**, 1806
	$H_{ox}H$	2090, 2075, 1975, **1953**, 1809
	H_{ox}-CO	2090, 2075, **2015**, 1973, 1967, 1806
	H_{red}’	ND, 2062, 1960, **1937**, 1787
	$H_{red}H^+$	2054, 2040, 1921, **1899**, 1801
	$H_{sred}H^+$-like	2042, 2022, **1893**, 1878, 1781
**Truncated *Ca*HydA1**	H_{ox}	2082, 2071, 1981, **1951**, 1797
	H_{ox}^{PDT}	2083, 2072, 1981, **1953**, 1803
	$H_{red}H^+$	2054, 2040, 1921, **1899**, 1801
	H_{hyd}-like	ND, ND, 1996, ND, **1867**, **1860**

Table S8. *Ca*HydA1 [FeFe] hydrogenase variants EPR simulation parameter values. [a]Linewidths were $\sigma_{1,2,3}$ =7.9,7.9,7.2 MHz [b]An axial g-matrix is assumed. Linewidths were $\sigma_{1,2,3}$= 25,13,13 MHz

State	Protein		Component fraction	g_1	g_2	g_3	g_{iso}
H_{ox} [a]	WT	Comp 1	F=0.7	2.096	2.0395	2.0005	2.0453
		Comp 2	F=0.3	2.0942	2.0355	1.9975	2.0424
	S75	Comp 1	F=1	2.0930	2.0342	1.996	2.0411
	D127	Comp 1	F=0.25	2.0965	2.0392	2.0005	2.0454
		Comp 2	F=0.75	2.0942	2.0358	1.9975	2.0425
	S208	Comp 1	F=1	2.0946	2.0357	1.9975	2.0426
H_{ox}-CO [b]	WT	Comp 1	F=0.9	2.075	2.0069	2.0069	2.0296
		Comp 2	F=0.1	2.0225	2.0069	2.0069	2.0121
	S75	Comp 1	F=1	2.02	2.008	2.008	2.012
	D127	Comp 1	F=1	2.022	2.009	2.009	2.0133
	S208	Comp 1	F=1	2.022	2.0095	2.0095	2.0137
Apo-protein	Apo	$[2Fe\text{-}2S]^+$	F=1	2.025	1.935	1.935	1.965

Supplementary References

[1] V. Fourmond, T. Lautier, C. Baffert, F. Leroux, P.-P. Liebgott, S. Dementin, M. Rousset, P. Arnoux, D. Pignol, I. Meynial-Salles, P. Soucaille, P. Bertrand, C. Léger, *Anal. Chem.* **2009**, *81*, 2962-2968.
